# Impact of kidney dysfunction on outcomes in peripheral artery disease: A multi-database cohort study

**DOI:** 10.1016/j.isci.2026.115681

**Published:** 2026-04-10

**Authors:** Heng Wang, Chaonan Fan, Keyi Fan, Yijie Ning, Ziyan Wang, Yaling Li, Runze Chang, Jianhua Jiang, Jiang Han, Yongbin Shi, Yuwen Wang, Shule Wang, Yimiao Wei, Keyang Xu, Yun Zhou, Guoping Zheng

**Affiliations:** 1 Centre for Transplant and Renal Research, Westmead Institute for Medical Research, The University of Sydney, Sydney, NSW, Australia; 2 Faculty of Medicine and Health, The University of Sydney, Camperdown, NSW, Australia; 3 Department of Nephrology, The Second Hospital of Shanxi Medical University, Taiyuan, Shanxi, China; 4 Department of Vascular Surgery, The Second Hospital of Shanxi Medical University, Taiyuan, Shanxi, China; 5 Department of Endovascular Surgery, The First Affiliated Hospital of Zhengzhou University, Zhengzhou, Henan, China; 6 The Department of Vascular Surgery, Beijing Anzhen Hospital, Capital Medical University, Beijing, China; 7 Neuroscience Institute, JFK University Medical Center, Edison, NJ, USA; 8 Department of Obstetrics and Gynecology, The Eighth Affiliated Hospital of Southern Medical University, Foshan, Guangdong, China; 9 Faculty of Chinese Medicine and State Key Laboratory of Quality Research in Chinese Medicine, Macau University of Science and Technology, Macau, China; 10 Department of Nephrology, The First Hospital of Shanxi Medical University, Taiyuan, Shanxi, China

**Keywords:** Health sciences, Medicine, Medical specialty, Cardiovascular medicine, Nephrology

## Abstract

Peripheral artery disease (PAD) and kidney dysfunction frequently coexist, worsening clinical outcomes. This study aimed to clarify the relationship between PAD and kidney dysfunction, including their co-occurrence, impact on outcomes, and potential causal links. Analyzing GBD 2021 data, we identified a global PAD prevalence of 1,326.45 per 100,000, with renal impairment as a primary mortality driver. CDC WONDER data ranked renal disease 8th in PAD-related deaths (AAMR 0.22). In MIMIC-IV (*n* = 2,133), AKI significantly raised 30-day mortality (21.01% vs. 7.23%; HR = 3.21), while CKD stage 4–5 increased 365-day mortality risk (HR = 2.20). 2H-SXMU clinical cohort (*n* = 200) showed lower eGFR (74.9 vs. 84.6 mL/min/1.73 m^2^) associated with increased major adverse limb events. Mendelian randomization confirmed a negative causal effect of PAD on eGFR (OR = 0.997, *p* = 1.75e^−3^), with no reverse causation found. These findings demonstrate that PAD contributes to declining renal function, highlighting the need for integrated nephrology vascular care and routine renal monitoring in PAD management.

## Highlights

• Global and national data show kidney dysfunction as one of the primary drivers of PAD mortality
• AKI and CKD stages 4–5 significantly elevate short- and long-term PAD mortality
• Lower eGFR is linked to an increased risk of major adverse limb events in 2H-SXMU cohorts
• Findings support integrated care and routine renal monitoring for PAD management

## Graphical abstract

**Figure:**
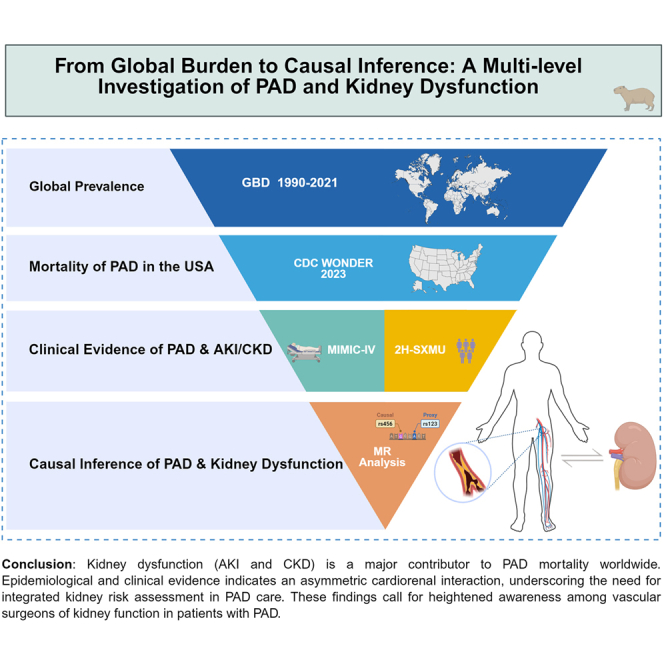

## Introduction

Kidney dysfunction has emerged as a major global public health burden, currently affecting over 850 million individuals worldwide and contributing to millions of deaths annually, and the figure continues to rise.^1^^,^^2^^,^^3^ Over the past half-century, kidney dysfunction has been broadly categorized into two clinical categories: acute kidney injury (AKI) and chronic kidney disease (CKD), both of which may ultimately progress to renal fibrosis and kidney failure.^4^ AKI is defined by an abrupt decline in glomerular filtration rate (GFR), often accompanied by elevated serum creatinine (Scr) levels, with oliguria, or anuria being hallmark clinical manifestations.^5^ Each episode of AKI carries a high risk of mortality and is associated with long-term adverse outcomes, including cardiovascular complications, progression to CKD, and end-stage kidney disease (ESKD).^6^^,^^7^ Population growth, aging, and the increasing prevalence of diabetes, cardiovascular disease, and hypertension are the predominant risk factors for CKD. Among them, the coexistence of diabetes, atherosclerosis, and CKD contributes to microvascular complications, peripheral artery disease (PAD), coronary artery disease, and cerebrovascular events.^8^^,^^9^^,^^10^

Previous studies have identified CKD as an independent risk factor for the progression of PAD.^11^^,^^12^^,^^13^ Beyond traditional risk factors such as diabetes and hypertension, CKD-specific pathophysiological mechanisms, including vascular calcification, chronic inflammation, electrolyte imbalance, uremic toxins, and microvascular dysfunction, contribute substantially to the increased risk of PAD in individuals with CKD.^14^^,^^15^^,^^16^^,^^17^ Conversely, both symptomatic and asymptomatic PAD have been independently associated with an elevated risk of CKD progression and the development of ESKD.^9^^,^^18^ These findings highlight a bidirectional and mutually reinforcing relationship between CKD and PAD, forming a complex comorbidity pattern with intertwined pathophysiology and accelerated clinical deterioration. In current clinical practice, the management of patients with CKD often includes vigilance for PAD-related complications such as lower limb ischemia, foot ulceration, critical limb ischemia, and amputation.^19^^,^^20^ However, among patients receiving specialized care for PAD, there remains a lack of consistent attention to kidney function, particularly regarding preoperative evaluation, in-hospital monitoring, and long-term post-discharge follow-up.^21^^,^^22^

In this study, we integrated global, national, clinical, and genetic data to examine the relationship between PAD and kidney dysfunction. By combining epidemiological analyses and clinical cohorts with Mendelian randomization (MR) analysis as an exploratory genetic approach, we aimed to identify the burden and prognostic impact of coexisting kidney dysfunction in patients with PAD.

## Results

### Global trends in the disease burden of patients with PAD

**Figure 1:**
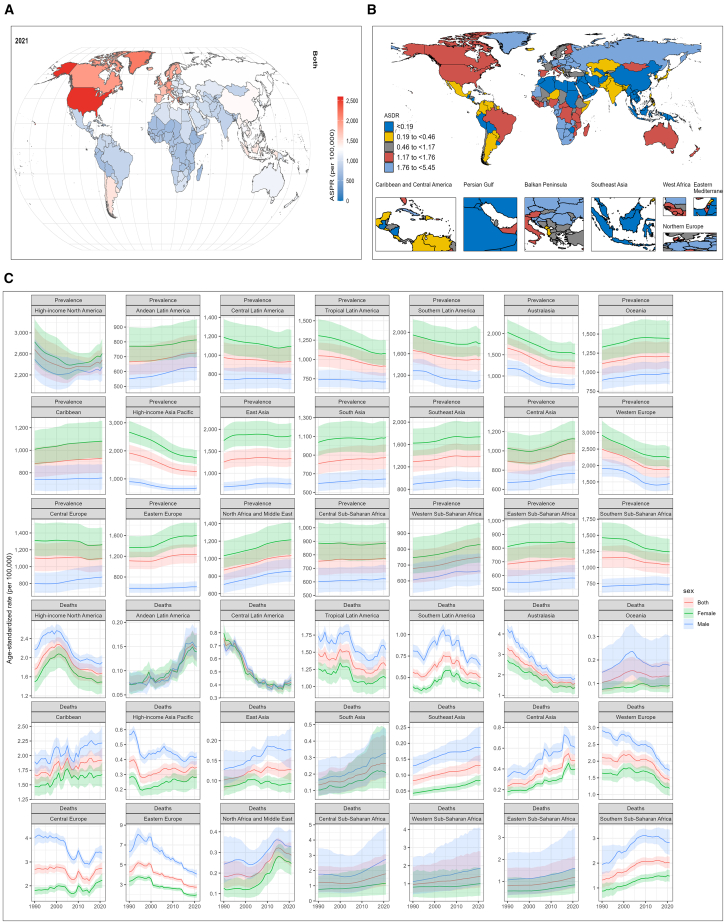
Global burden of peripheral artery disease (PAD) (A) Age-standardized prevalence rate (ASPR) of PAD in 2021. (B) Age-standardized death rate (ASDR) of PAD in 2021. (C) Trends in ASPR, age-standardized death rate (ASDR), and age-standardized disability-adjusted life years rate (ASDALYR) for PAD across 21 global burden of disease (GBD) regions from 1990 to 2021.

Age-standardized prevalence rate (ASPR), age-standardized death rate (ASDR), and age-standardized disability-adjusted life year rate (ASDALYR) for PAD in 2021 are presented in Figures 1 and S4. Globally, the ASPR for PAD was 1,326.45 (95% uncertainty interval [UI]: 1,153.78–1,526.53), with values of 953.49 (830.78–1,098.16) for males and 1,643.00 (1,425.90–1,893.27) for females (Figure 1A). The global ASDR was 0.85 (0.75–0.93), higher in males (0.98) than females (0.73) (Figure 1B). Countries with the highest ASPR included the USA, Denmark, Greenland, Luxembourg, and Greece, while the highest ASDRs were observed in Barbados, Belarus, Poland, Ukraine, and Hungary.

From 1990 to 2021, global ASPR, ASDR, and ASDALYR for PAD exhibited overall declining trends (Figure S1). Temporal trends in ASPR and ASDR across the 21 GBD regions are shown in Figure 1C. Notably, increasing ASPR trends were observed in Andean Latin America, Oceania, the Caribbean, Southeast Asia, Eastern Europe, North Africa and the Middle East, and Western Sub-Saharan Africa. Regions with increasing ASDR trends included Andean Latin America, the Caribbean, parts of Asia, and Africa. In the five socio-demographic index (SDI) strata, ASPR, ASDR, and ASDALYR tended to increase in regions with lower SDI levels (Figure S1).

Finally, we assessed the correlation between ASPR/ASDR and SDI across different countries and regions in 2021 (Figure S1). Among the 21 GBD regions, ASPR was positively correlated with SDI (*R* = 0.22, *p* < 0.001), and ASDR also showed a significant positive correlation with SDI (*R* = 0.46, *p* < 0.001) (Figures S2A and S2B). Across 204 countries and territories, both male and female ASPR values were positively associated with SDI. Female ASDR also showed a significant positive correlation with SDI, whereas the correlation between male ASDR and SDI was not statistically significant (Figures S2C–S2F).

### Kidney dysfunction as a leading global risk factor for PAD-related mortality

**Figure 2:**
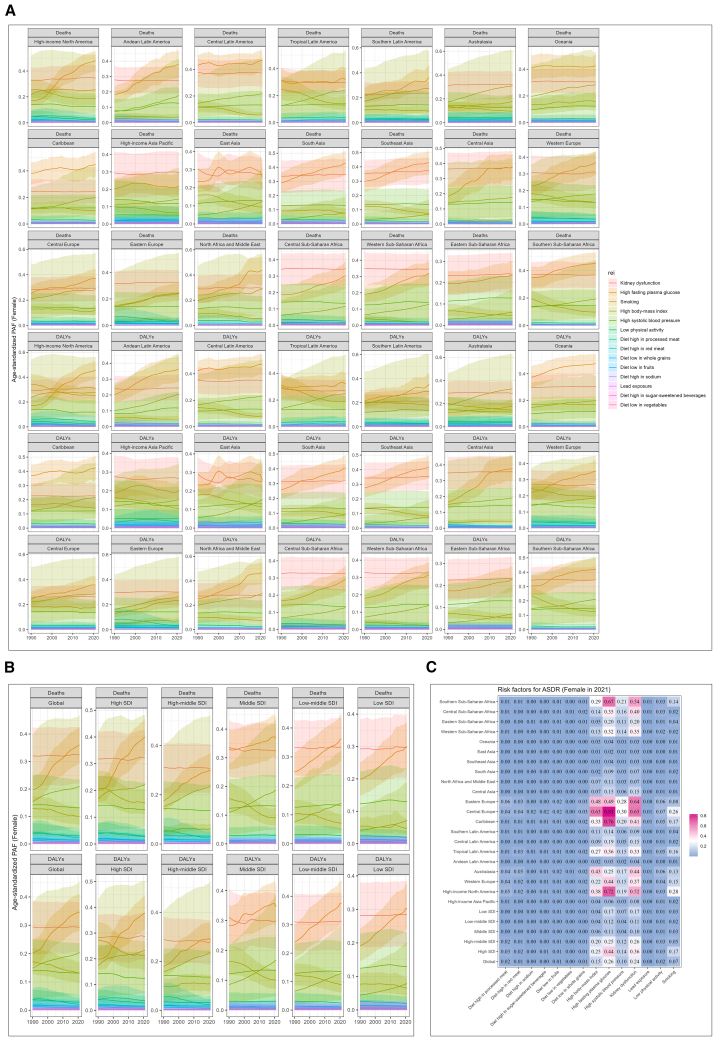
Global risk factors for peripheral artery disease (PAD) in female patients (A) Population attributable fractions (PAFs) of risk factors for PAD-related deaths in females across 21 global burden of disease (GBD) regions from 1990 to 2021. (B) PAFs of PAD-related deaths in females globally and across five sociodemographic index (SDI) regions from 1990 to 2021. (C) Age-standardized death rates (ASDR) of PAD risk factors in females globally in 2021.

We further examined the global population attributable fraction (PAF) of risk factors for PAD-related mortality, stratified by sex (Figures 2 and S3). Notably, among both females and males, mortality attributable to kidney dysfunction remained consistently high and stable over time.

Figure 2 presents the spatiotemporal distribution of risk factors contributing to PAD-related deaths in females. Across the 21 GBD regions, kidney dysfunction ranked among the top causes of PAD-related mortality and was the leading attributable risk factor in several regions, including Australia, high-income Asia Pacific, East Asia, Eastern Europe, Central Sub-Saharan Africa, Western Sub-Saharan Africa, and Eastern Sub-Saharan Africa (Figure 2A). Among the five SDI strata, kidney dysfunction was the primary cause of PAD-related mortality in high-, middle-, and low-SDI regions (Figure 2B). Figure 2C shows the ASDR attributable to kidney dysfunction in 2021 at the global level, across GBD regions, and among SDI strata for females.

Figure S3 depicts the spatiotemporal trends of PAD-related mortality risk factors in males. In contrast to females, the predominant contributors to male PAD mortality included high fasting plasma glucose, kidney dysfunction, and smoking. Like the trend observed in females, the PAF for kidney dysfunction in males remained consistently elevated across regions and time points, with minimal variation.

### The mortality risk of PAD combined with kidney dysfunction in the USA

From 1999 to 2023, a total of 179,794 deaths were recorded in the USA with PAD listed as the underlying cause of death. Among these, CKD was identified as a contributing cause of death in 17,397 cases (Table S2), while AKI was identified as a contributing cause of death in 6,356 cases (Table S3). We subsequently evaluated the 15 leading causes of death among decedents with PAD. In the aggregated data from 1999 to 2020, when PAD was listed as a contributing cause of death, nephritis, nephrotic syndrome, and nephrosis (N00–N07, N17–N19, and N25–N27) ranked as the eighth leading cause of death, with an age-adjusted mortality rate (AAMR) of 0.22 (95% confidence interval [CI], 0.22–0.23) (Figure S4A). In the 2024 data, nephritis, nephrotic syndrome, and nephrosis (N00–N07, N17–N19, and N25–N27) accounted for 614 deaths among patients with PAD (Figure S4B). We next examined temporal trends in AAMR among patients with PAD combined with CKD (Figure S4C) and PAD combined with AKI (Figure S4E). Differences in AAMR were observed across the USA census regions, with the South and Midwest consistently exhibiting higher AAMRs and an overall upward trend, suggesting a gradually increasing mortality burden. In addition, patients with PAD combined with CKD or AKI demonstrated similar geographic patterns of mortality risk. At the state level, mortality burden among patients with PAD combined with CKD and those with PAD combined with AKI was unevenly distributed across the USA. Several Southern states (e.g., Texas) exhibited both high numbers of deaths and elevated AAMRs, whereas some Western states (e.g., California) showed relatively lower AAMRs despite having a high number of deaths. States in the Midwest displayed a wide dispersion of AAMRs across different levels of death counts (Figures S4D and S4F).

**Figure 3:**
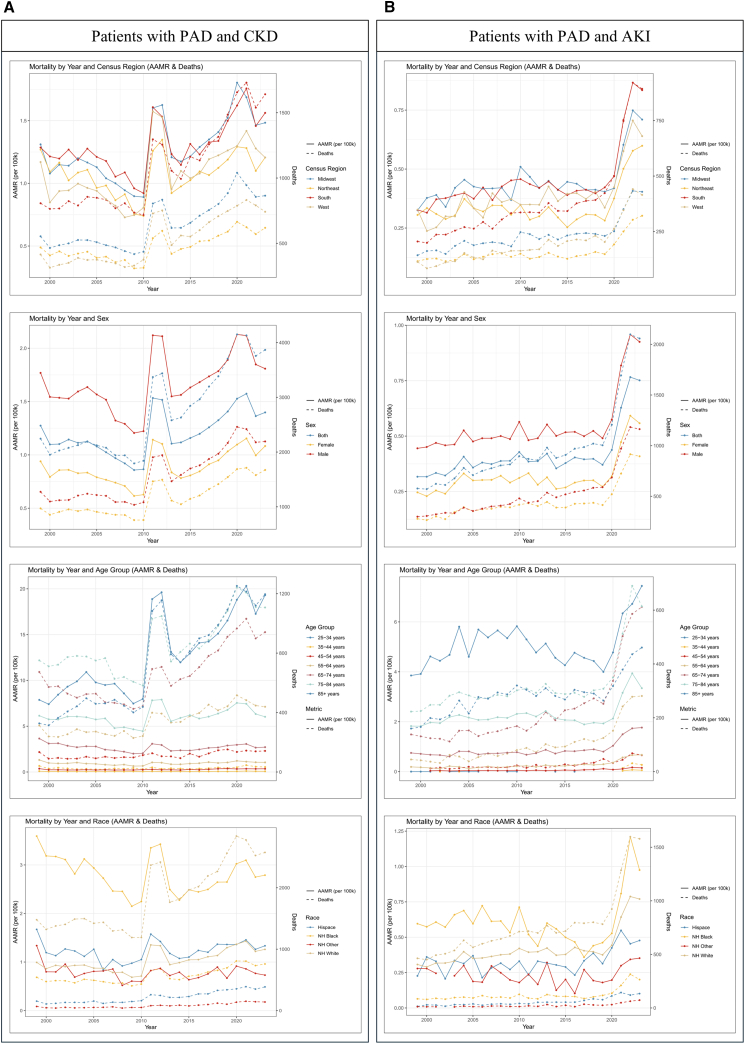
Mortality among patients with PAD and kidney dysfunction in the USA (A) Age-adjusted mortality rates (AAMRs) and number of deaths among patients with PAD and CKD. (B) AAMRs and number of deaths among patients with PAD and AKI.

Mortality analyses were conducted separately for patients with PAD and CKD (Figure 3A) and patients with PAD and AKI (Figure 3B**)**. Overall, individuals residing in the Midwest and South regions, males, older adults, and non-Hispanic Black populations demonstrated relatively higher AAMRs and greater numbers of deaths.

Furthermore, we quantified AAMRs among patients with PAD combined with CKD and AKI across the US states from 1999 to 2023. Despite the presence of some missing values, overall AAMRs exhibited varying degrees of increase over time, with substantial interstate heterogeneity. In comparison, the absolute AAMR levels among patients with AKI were consistently lower than those observed among patients with CKD (Figure S5).

### Clinical characteristics of the study population in MIMIC-IV database

The patient selection process is illustrated in Figure S6. A total of 9,750 individuals diagnosed with PAD and with only their first hospital admission recorded were initially identified from the Medical Information Mart for Intensive Care IV (MIMIC-IV) database. After applying exclusion criteria, 2,133 patients were included in the final analysis. Among them, 689 (32.30%) had comorbid CKD, whereas 1,444 (67.70%) did not (Table 1). After propensity score matching (PSM), baseline characteristics between the two groups were well balanced, with most variables demonstrating *p* values >0.05 and standardized mean differences (SMDs) <0.1. A total of 371 matched pairs of patients with PAD were included in the subsequent analyses (Table 1; Figure S7). In the final cohort, 1,571 patients developed AKI during the index hospitalization, whereas 562 did not.

**Table 1:** Comparison of baseline clinical characteristics in PAD patients with and without CKD before and after propensity score matching (PSM)

	Before PSM (*n* = 2133)					After PSM (*n* = 742)				
	Total	without CKD (*n* = 1444)	with CKD (*n* = 689)	*P*	SMD	total	without CKD (*n* = 371)	with CKD (*n* = 371)	*P*	SMD
**Demographics**										

Age	71.00 (62.00, 79.00)	70.00 (61.00, 78.00)	75.00 (66.00, 82.00)	<0.001	0.404	73.00 (66.00, 81.00)	72.00 (66.00, 80.00)	74.00 (65.50, 81.00)	0.323	0.022
Gender, n (%)				0.021					0.940	
Female	906 (42.48)	638 (44.18)	268 (38.90)		−0.108	303 (40.84)	151 (40.70)	152 (40.97)	–	0.005
Male	1,227 (57.52)	806 (55.82)	421 (61.10)		0.108	439 (59.16)	220 (59.30)	219 (59.03)	–	−0.005
Race, *n* (%)				<0.001					0.263	
African	114 (5.34)	47 (3.25)	67 (9.72)		0.218	42 (5.66)	19 (5.12)	23 (6.20)	–	0.045
Other	577 (27.05)	388 (26.87)	189 (27.43)		0.013	207 (27.9)	95 (25.61)	112 (30.19)	–	0.100
Caucasian	1,442 (67.6)	1,009 (69.88)	433 (62.84)		−0.145	493 (66.44)	257 (69.27)	236 (63.61)	–	−0.118

**Vital signs**										

Heart rate	82.00 (72.00, 94.00)	82.00 (72.00, 94.00)	82.00 (71.00, 94.00)	0.908	−0.000	83.00 (73.00, 96.00)	83.00 (71.00, 96.00)	83.00 (74.00, 96.00)	0.520	0.025
SpO2	98.00 (95.00, 100.00)	98.00 (96.00, 100.00)	98.00 (95.00, 100.00)	0.006	−0.105	98.00 (95.00, 100.00)	98.00 (95.00, 100.00)	98.00 (95.00, 100.00)	0.151	0.012
SBP	124.00 (107.00, 141.00)	123.00 (107.00, 141.00)	124.00 (106.00, 143.00)	0.838	0.031	121.00 (105.25, 138.00)	120.00 (106.00, 137.00)	122.00 (105.00, 139.00)	0.795	0.012
DBP	64.00 (53.00, 76.00)	65.00 (54.00, 77.00)	61.00 (51.00, 75.00)	<0.001	−0.109	61.00 (52.00, 74.00)	61.50 (52.00, 74.00)	61.00 (52.00, 74.50)	0.914	−0.000
MBP	81.00 (70.00, 94.00)	82.00 (71.00, 94.00)	79.00 (68.00, 92.00)	<0.001	−0.102	79.00 (69.00, 91.00)	79.00 (69.00, 92.00)	79.00 (69.00, 91.00)	0.886	−0.023
RR	18.00 (15.00, 21.50)	17.00 (15.00, 21.00)	18.00 (16.00, 22.00)	0.002	0.123	18.00 (15.00, 22.00)	17.00 (14.00, 22.00)	18.00 (15.00, 22.00)	0.116	0.054
SOFA	4.00 (2.00, 7.00)	4.00 (2.00, 6.00)	5.00 (3.00, 8.00)	<0.001	0.443	5.00 (3.00, 7.00)	4.00 (2.00, 7.00)	5.00 (3.00, 7.00)	0.123	0.069

**Laboratory findings**										

RBC	3.65 (3.16, 4.15)	3.75 (3.25, 4.25)	3.47 (2.98, 3.91)	<0.001	−0.410	3.57 (3.13, 4.06)	3.52 (3.09, 4.05)	3.61 (3.14, 4.06)	0.377	0.057
WBC	9.90 (7.30, 13.40)	10.00 (7.30, 13.40)	9.70 (7.30, 13.30)	0.303	−0.008	9.70 (7.23, 13.67)	9.90 (7.30, 13.75)	9.50 (7.20, 13.60)	0.457	0.033
Platelet	197.00 (151.00, 259.00)	198.00 (153.00, 261.00)	196.00 (147.00, 255.00)	0.248	−0.080	198.00 (154.00, 260.00)	195.00 (154.00, 255.00)	203.00 (154.00, 264.00)	0.475	0.036
Glucose	126.00 (102.00, 161.00)	121.00 (101.00, 155.25)	132.00 (106.00, 178.00)	<0.001	0.199	126.00 (101.00, 164.00)	126.00 (100.00, 163.50)	126.00 (101.00, 164.50)	0.524	0.054

**Comorbidities**										

Heart failure, *n* (%)				<0.001					0.184	
No	1,254 (58.79)	991 (68.63)	263 (38.17)		−0.627	334 (45.01)	158 (42.59)	176 (47.44)	–	0.097
Yes	879 (41.21)	453 (31.37)	426 (61.83)		0.627	408 (54.99)	213 (57.41)	195 (52.56)	–	−0.097
AS, *n* (%)				<0.001					0.607	
No	1,174 (55.04)	852 (59.00)	322 (46.73)		−0.246	389 (52.43)	198 (53.37)	191 (51.48)	–	−0.038
Yes	959 (44.96)	592 (41.00)	367 (53.27)		0.246	353 (47.57)	173 (46.63)	180 (48.52)	–	0.038
Diabetes, *n* (%)				<0.001					0.366	
No	1,375 (64.46)	1,039 (71.95)	336 (48.77)		−0.464	454 (61.19)	233 (62.80)	221 (59.57)	–	−0.066
Yes	758 (35.54)	405 (28.05)	353 (51.23)		0.464	288 (38.81)	138 (37.20)	150 (40.43)	–	0.066
Hypertension, *n* (%)				<0.001					0.594	
No	1,058 (49.6)	448 (31.02)	610 (88.53)		1.805	580 (78.17)	287 (77.36)	293 (78.98)	–	0.040
Yes	1,075 (50.4)	996 (68.98)	79 (11.47)		−1.805	162 (21.83)	84 (22.64)	78 (21.02)	–	−0.040

**Intervention**										

MV, *n* (%)				0.023					0.496	
No	314 (14.72)	230 (15.93)	84 (12.19)		−0.114	88 (11.86)	47 (12.67)	41 (11.05)	–	−0.052
Yes	1,819 (85.28)	1,214 (84.07)	605 (87.81)		0.114	654 (88.14)	324 (87.33)	330 (88.95)	–	0.052

The sample size (*n*) represents individual human patients. The initial cohort before PSM included a total of 2,133 patients, which was subsequently matched to a final cohort of 742 patients. Continuous variables are expressed as medians with interquartile ranges (Q1, Q3), and categorical variables are presented as exact numbers (n) and percentages (%). Statistical differences between the non-CKD and CKD groups were assessed using the Mann-Whitney *U* test for continuous variables and the Pearson’s chi-squared test for categorical variables. The standardized mean difference (SMD) was calculated to evaluate the balance of baseline covariates between the two groups; an SMD close to 0 (typically <0.1) indicates adequate matching balance. Exact *p* values are provided; *p* < 0.001 indicates values below the limit of precision.

PAD, peripheral arterial disease; CKD, chronic kidney disease; PSM, propensity score matching; SOFA, sequential organ failure assessment; SBP, systolic blood pressure; DBP, diastolic blood pressure; MBP, mean blood pressure; RR, respiratory rate; RBC, red blood cell count; WBC, white blood cell count; SpO_2_, peripheral capillary oxygen saturation; MV, mechanical ventilation; AS, atherosclerosis; SMD, standardized mean difference; Q_1_ and Q_3_, first and third quartiles.

Patients with PAD + CKD group were older (median age 75 vs. 70 years, *p* < 0.001), had higher sequential organ failure assessment (SOFA) scores (5 vs. 4, *p* < 0.001), and exhibited significantly higher rates of heart failure (61.83% vs. 31.37%, *p* < 0.001), atherosclerosis (53.27% vs. 41.00%, *p* < 0.001), and diabetes mellitus (51.23% vs. 28.05%, *p* < 0.001). Racial distribution also differed between groups, with a higher proportion of non-Caucasian individuals in the PAD with CKD group (37% vs. 30%, *p* < 0.001). Vital signs showed significant differences, including lower diastolic blood pressure (DBP) (61 mmHg vs. 65 mmHg, *p* < 0.001), mean arterial pressure (MAP) (79 mmHg vs. 82 mmHg, *p* < 0.001), and higher respiratory rate (RR) (18 vs. 17 breaths per minute, *p* = 0.002) in the PAD with CKD group. Additionally, mechanical ventilation (MV) was more frequently used in PAD with CKD group (87.81% vs. 84.07%, *p* = 0.023). Laboratory measurements also revealed statistically significant differences, including lower red blood cell count (3.47 vs. 3.75 × 10^6^/μL, *p* < 0.001) and higher blood glucose levels (132 vs. 121 g/dL, *p* < 0.001) in the PAD with CKD group.

### Impact of kidney dysfunction on mortality in patients with PAD in MIMIC-IV database

**Figure 4:**
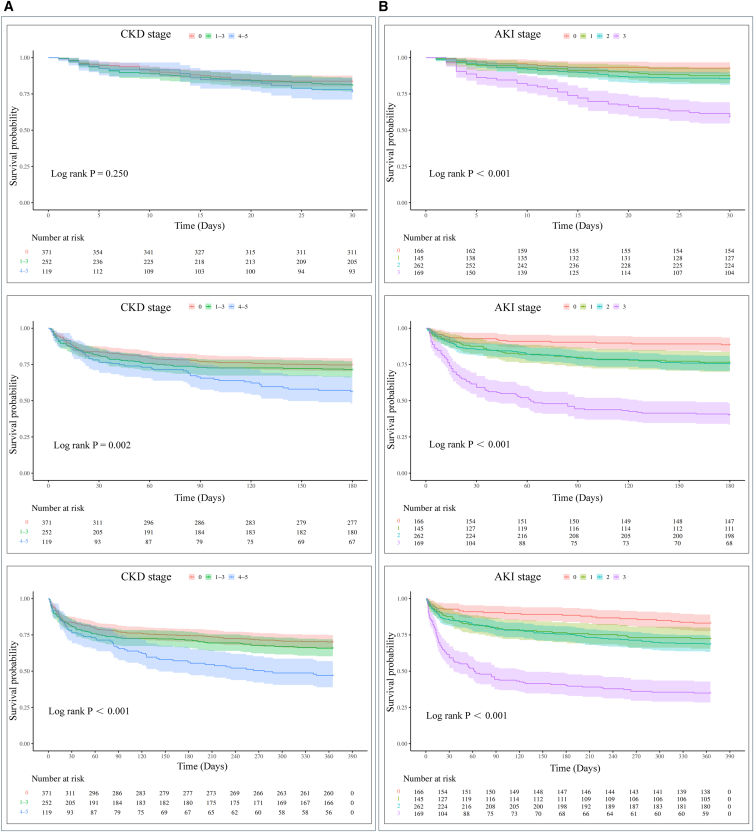
Short- and long-term survival in peripheral artery disease (PAD) patients stratified by kidney function Kaplan-Meier survival curves for all-cause mortality at 30 days, 180 days, and 365 days in PAD patients, stratified by chronic kidney disease (CKD) stage (A) and acute kidney injury (AKI) stage (B). The analysis was based on a total cohort of 742 individual human patients; the exact number of patients at risk at specific time points is provided in the tables below each plot. The shaded bands around the survival curves represent the 95% confidence interval (CI). Statistical comparisons among different stages were performed using the log rank test. Exact *p* values are displayed within each figure; *p* < 0.001 indicates values below the limit of precision.

Kaplan-Meier survival analyses were performed to evaluate the impact of kidney dysfunction on all-cause mortality at 30, 180, and 365 days after hospital admission in patients with PAD (Figure 4; Figure S8). At 365 days, mortality was 40.16% in PAD patients with CKD, compared to 29.92% in those without CKD. Notably, at 30 days, PAD patients with AKI had a markedly higher mortality rate (21.01%) than those without AKI (7.23%).

We first assessed the impact of comorbid CKD or AKI on survival outcomes in PAD patients (Figure S8). Within 30 days of admission, CKD was not significantly associated with decreased survival (*p* = 0.170; HR = 1.264; and 95% CI: 0.903–1.771), whereas AKI was strongly associated with early mortality (*p* < 0.001; HR = 3.213; and 95% CI: 1.777–5.809). As follow-up progressed, CKD was associated with significantly increased mortality at 180 days (*p* = 0.021; HR = 1.369; and 95% CI: 1.047–1.790), while the risk associated with AKI remained elevated (*p* < 0.001; HR = 3.445; and 95% CI: 2.152–5.517). At 365 days, PAD patients with CKD continued to show reduced survival (*p* = 0.005; HR = 1.415; and 95% CI: 1.107–1.810), and those with AKI maintained a significantly higher risk of death (*p* < 0.001; HR = 2.816; and 95% CI: 1.902–4.170). These findings suggest that CKD primarily contributes to long-term adverse outcomes in PAD, whereas AKI exerts a substantial impact on short-term prognosis.

We next examined the influence of CKD and AKI stages on survival (Figure 4). At 30 days, CKD stage was not significantly associated with survival (*p* = 0.250), whereas AKI stage showed a strong correlation with early mortality (*p* < 0.001). With prolonged follow-up, both CKD and AKI stage were positively associated with mortality at 180 and 365 days. Specifically, at 365 days, mortality was 34.13% in PAD patients with CKD stage 1–3 and 52.94% in those with CKD stage 4–5. At 30 days, mortality among patients with AKI stage 1 was 12.41%, stage 2 was 14.50%, and stage 3 was 38.46%. These results indicate that the severity of kidney dysfunction is directly associated with increased mortality risk in patient with PAD.

To identify risk factors associated with mortality in patients with PAD patients, we first compared the clinical characteristics of those who survived versus those who died within 180 days (Table S4). Compared with survivors, nonsurvivors were older and had higher SOFA scores, heart rate, respiratory rate, and lower SpO_2_. They also had a higher prevalence of heart failure and were more likely to receive MV and continuous renal replacement therapy (CRRT). Notably, CKD was present in 47% of survivors and 57% of nonsurvivors, with 24% of the latter having CKD stage 4–5. Additionally, AKI was observed in 72% of survivors and 91% of nonsurvivors, of whom 46% had AKI stage 3. Univariate Cox regression analysis for 180-day mortality identified 14 variables significantly associated with increased risk, including age, SOFA score, heart rate, SpO_2_, systolic blood pressure, RR, heart failure, AKI, white blood cell (WBC), blood urea nitrogen (BUN), Scr, MV, CRRT, and estimated glomerular filtration rate (eGFR) (Table S5). The corresponding analysis for 365-day mortality is shown in Table S6.

Time-dependent Cox regression analysis demonstrated that CKD was significantly associated with 180-day and 365-day mortality patients with PAD, but not with 30-day mortality (Table 2). When CKD status (presence vs. absence) was used as a predictor of 365-day mortality, the hazard ratios (HRs) were as follows: model 1: HR = 1.42 (95% CI: 1.11–1.81), *p* = 0.006; model 2: HR = 1.38 (95% CI: 1.08–1.76), *p* = 0.010; and model 3: HR = 1.44 (95% CI: 1.12–1.84), *p* = 0.004. When CKD stage was modeled, patients with CKD stage 4–5 had significantly increased 365-day mortality: model 1: HR = 1.96 (95% CI: 1.43–2.66), *p* < 0.001; model 2: HR = 2.18 (95% CI: 1.59–2.97), *p* < 0.001; and model 3: HR = 2.20 (95% CI: 1.61–3.02), *p* < 0.001. A similar trend was observed for 180-day mortality. Overall, more advanced CKD stages and longer disease duration were associated with higher mortality risk in patients with PAD.

**Table 2:** Multivariable time-varying Cox proportional hazards regression analysis of the association between CKD and all-cause mortality in PAD patients

Variables	Model 1		Model 2		Model 3	
	HR (95% CI)	P	HR (95% CI)	P	HR (95% CI)	P
Day 30						
CKD						
No	1.00 (reference)	–	1.00 (reference)	–	1.00 (reference)	–
Yes	1.26 (0.90–1.77)	0.173	1.22 (0.87–1.71)	0.242	1.23 (0.88–1.73)	0.232
CKD stage						
0	1.00 (reference)	–	1.00 (reference)	–	1.00 (reference)	–
1-3	1.18 (0.81–1.72)	0.401	1.06 (0.73–1.55)	0.756	1.09 (0.74–1.60)	0.669
4-5	1.45 (0.93–2.27)	0.103	1.65 (1.05–2.58)	0.030	1.58 (1.01–2.48)	0.050
Day 180						
CKD						
No	1.00 (reference)	–	1.00 (reference)	–	1.00 (reference)	–
Yes	1.37 (1.05–1.79)	0.022	1.33 (1.02–1.75)	0.035	1.38 (1.05–1.81)	0.021
CKD stage						
No	1.00 (reference)	–	1.00 (reference)	–	1.00 (reference)	–
1–3	1.16 (0.85–1.58)	0.345	1.06 (0.78–1.45)	0.693	1.11 (0.81–1.52)	0.502
4–5	1.83 (1.30–2.56)	<0.001	2.05 (1.46–2.88)	<0.001	2.02 (1.43–2.85)	<0.001
Day 365						
CKD						
No	1.00 (reference)	–	1.00 (reference)	–	1.00 (reference)	–
Yes	1.42 (1.11–1.81)	0.006	1.38 (1.08–1.76)	0.010	1.44 (1.12–1.84)	0.004
CKD stage						
No	1.00 (reference)	–	1.00 (reference)	–	1.00 (reference)	–
1-3	1.18 (0.89–1.56)	0.255	1.09 (0.82–1.44)	0.570	1.14 (0.86–1.52)	0.374
4-5	1.96 (1.43–2.66)	<0.001	2.18 (1.59–2.97)	<0.001	2.20 (1.61–3.02)	<0.001

The analysis included 742 matched human patients. Time-varying Cox proportional hazards models were used to calculate hazard ratios (HR) and 95% confidence intervals (CI) at days 30, 180, and 365. Patients without CKD or with CKD stage 0 served as reference groups (HR = 1.00). Model descriptions: model 1 is unadjusted; model 2 is adjusted for gender, race, and age; and model 3 is adjusted for gender, race, age, heart failure, AKI, MV, heart rate, SpO_2_, SBP, RR, and WBC.

HR, hazard ratio; CI, confidence interval; CKD, chronic kidney disease; AKI, acute kidney injury; MV, mechanical ventilation; SBP, systolic blood pressure; RR, respiratory rate; WBC, white blood cell count; SpO_2_, peripheral capillary oxygen saturation.

**Figure 5:**
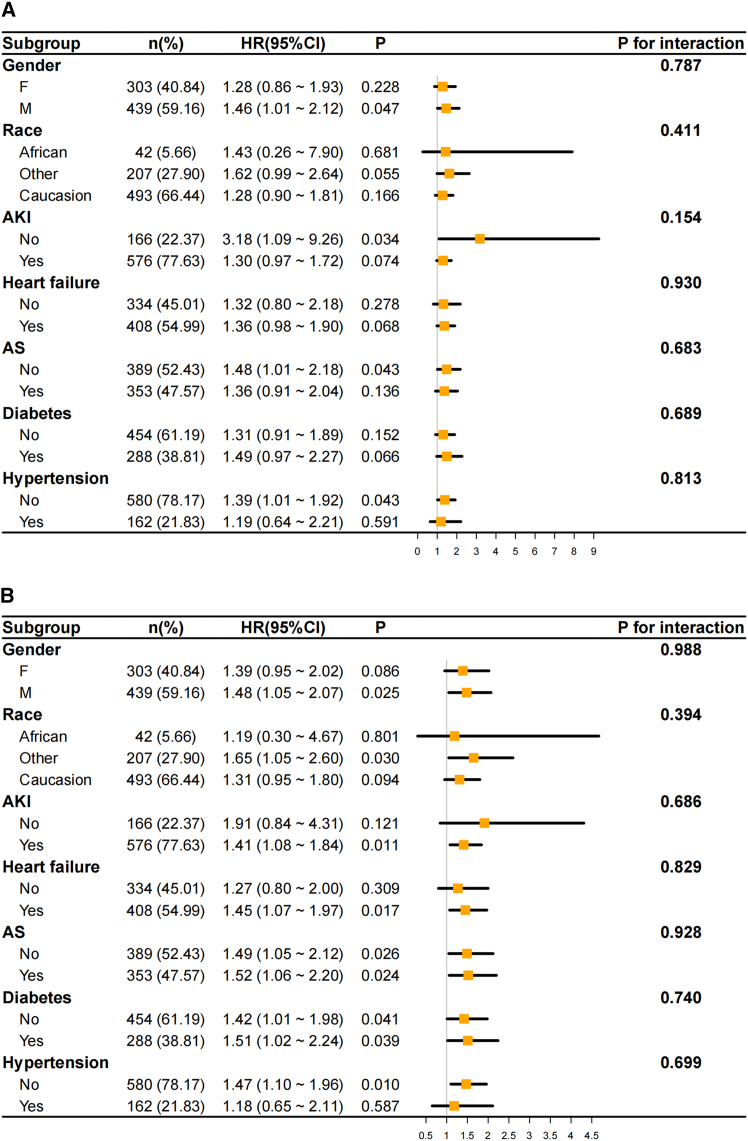
Subgroup analysis of chronic kidney disease (CKD)-associated mortality risk in patients with peripheral artery disease (PAD) Forest plots of subgroup analyses for the association between CKD and all-cause mortality in PAD patients at 180 days (A) and 365 days (B). The analysis was based on a total cohort of 742 individual human patients; the specific sample size (*n*) and percentage (%) for each subgroup are detailed within the figure. Subgroups were defined by sex, race, and comorbidities, including acute kidney injury (AKI), heart failure (HF), atherosclerosis (AS), diabetes mellitus (DM), and hypertension (HTN). Hazard ratios (HR, represented by orange squares) and 95% confidence intervals (CI, represented by horizontal black lines) were calculated using Cox proportional hazards regression models. The exact *p* values indicate the statistical significance of the HR within each specific stratum. The *P* for interaction was calculated to assess whether the effect of CKD on mortality differed significantly across the strata of each subgroup variable.

Finally, forest plots were generated to assess the association between CKD and mortality across various subgroups at 180 days (Figure 5A) and 365 days (Figure 5B**)**. Subgroup analyses were stratified by sex, race, and comorbidities including AKI, heart failure, atherosclerosis, diabetes, and hypertension. The effect of CKD on mortality was consistent across subgroups (P for interaction >0.05). Of note, male sex was associated with significantly higher mortality at both 180 days (*p* = 0.047) and 365 days (*p* = 0.025). At 180 days, the association between CKD and mortality was not significant in patients with comorbid AKI, atherosclerosis, or hypertension (*p* > 0.05). However, at 365 days, CKD remained significantly associated with mortality in patients with concomitant AKI or heart failure (*p* < 0.05). These findings highlight the complex interplay of comorbidities in modulating risk trajectories in PAD.

### MALE incidence and kidney function in PAD patients of the 2H-SXMU cohort

A total of 200 patients with PAD were included in the analysis and followed for 1 year after the index procedure. During follow-up, 83 patients experienced major adverse limb event (MALE), whereas 117 patients did not. Baseline characteristics of patients with and without MALE are summarized in Table 3. Patients in the MALE group were older than those in the non-MALE group (71.2 ± 9.2 vs. 68.6 ± 9.2 years, *p* = 0.045). Significant differences were also observed in smoking status between groups (*p* = 0.043). No significant differences were found with respect to sex distribution, body mass index, prevalence of type 2 diabetes, hypertension, ischemic heart disease, or hyperlipidemia (*p* > 0.05).

**Table 3:** Comparison of baseline clinical characteristics in patients with PAD from 2H-SXMU stratified by the occurrence of major adverse limb events (MALE)

Characteristics	Non-MALE (*n* = 117)	MALE (*n* = 83)	*p* value
Sex, *n* (%)			0.301
Male	92 (46%)	60 (30%)	–
Female	25 (12.5%)	23 (11.5%)	–
Age, mean ± sd	68.6 ± 9.2	71.2 ± 9.2	0.045
BMI, mean ± sd	23.20 ± 3.51	22.84 ± 3.05	0.444
Smoking, *n* (%)			0.043
Current	38 (19%)	39 (19.5%)	–
Ex-smoker	69 (34.5%)	34 (17%)	–
Non-smoker	10 (5%)	10 (5%)	–
Type 2 diabetes, *n* (%)			0.466
Yes	54 (27%)	34 (17%)	–
No	63 (31.5%)	49 (24.5%)	–
Hypertension, *n* (%)			0.177
Yes	65 (32.5%)	54 (27%)	–
No	52 (26%)	29 (14.5%)	–
Ischemic heart disease, *n* (%)			0.920
Yes	27 (13.6%)	20 (10.1%)	–
No	88 (44.4%)	63 (31.8%)	–
Hyperlipidemia, *n* (%)			0.800
Yes	34 (25.8%)	54 (40.9%)	–
No	16 (12.1%)	28 (21.2%)	–
Creatinine, median (IQR)	85.7 (75.6, 94.2)	92.1 (79.9, 109.4)	0.017
Urea, median (IQR)	6.5 (5.1, 8.1)	6.5 (5.2, 7.7)	0.428
eGFR, median (IQR)	84.6 (73.1, 93.5)	74.9 (62.6, 91.3)	0.003

The cohort comprises 200 individual human patients (83 with MALE and 117 without). Categorical variables are presented as *n* (%) and evaluated using Pearson’s chi-squared test or Fisher’s exact test. Continuous variables are presented as median with interquartile range (IQR), and compared using the Mann-Whitney *U* test, respectively. Exact *p* values are provided.

MALE, major adverse limb events; PAD, peripheral artery disease; BMI, body mass index; eGFR, estimated glomerular filtration rate; SD, standard deviation; IQR, interquartile range.

Regarding kidney function parameters, patients who developed MALE exhibited higher Scr levels (92.1 [79.9, 109.4] vs. 85.7 [75.6, 94.2] μmol/L, *p* = 0.017) and lower eGFR values (74.9 [62.6, 91.3] vs. 84.6 [73.1, 93.5] mL/min/1.73 m^2^, *p* = 0.003) compared with those without MALE. Urea levels were comparable between the two groups (*p* = 0.428). These differences are further illustrated in Figure S9.

When patients were stratified according to eGFR categories, a higher proportion of MALE was observed among patients with reduced kidney function, particularly in those with eGFR 30 to <60 mL/min/1.73 m^2^ (Table S7). Exploratory analyses indicated that this subgroup exhibited a significantly higher MALE proportion compared with the remaining cohort.

### Causal relationship between kidney dysfunction and PAD

**Figure 6:**
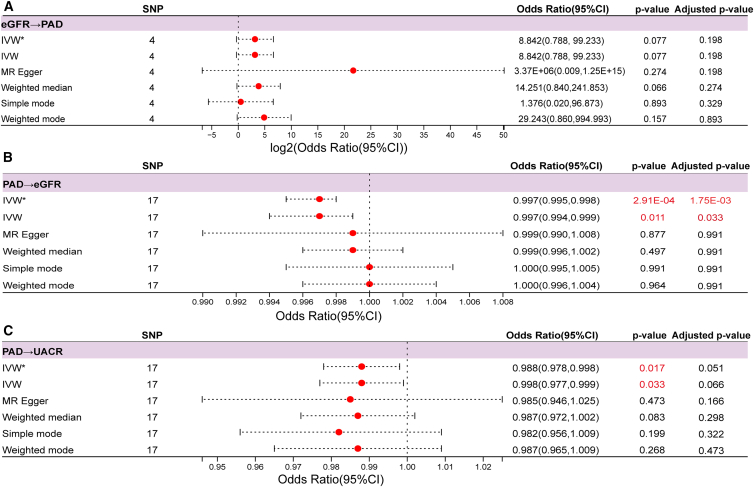
Forest plots of bidirectional causal relationships between kidney function markers and peripheral artery disease (PAD) based on Mendelian randomization analysis (A) Forest plot showing the causal effect of estimated glomerular filtration rate (eGFR) on PAD, expressed as odds ratio (OR) and 95% confidence interval (CI). (B) Forest plot showing the causal effect of PAD on eGFR. (C) Forest plot showing the causal effect of PAD on UACR. Instrumental variables were selected as single nucleotide polymorphisms (SNPs) significantly associated with exposures. The causal estimates were obtained using inverse variance weighted (IVW) methods, including both random effects (IVW) and fixed effects (IVW∗). The analysis utilized summary-level statistics from genome-wide association studies (GWASs) (detailed sample sizes for each dataset are provided in Table S9). Instrumental variables (the exact number of SNPs used is indicated in the figure) were selected based on their significant associations with the exposures. Point estimates for the odds ratios (OR) are represented by red dots, and their 95% confidence intervals (CI) are indicated by horizontal dashed lines. Exact *p* values and adjusted *p* values are displayed, with statistically significant values highlighted in red text.

Single nucleotide polymorphisms (SNPs) served as instrumental variables in the MR analysis, and the workflow for SNP selection is presented in Table S8. MR analyses revealed no significant causal effect of kidney function indicators on the risk of PAD. Specifically, using the inverse variance weighted (IVW) method, eGFR showed no causal association with PAD (odds ratio [OR] = 8.842; 95% CI: 0.788–99.233; and adjusted *p* = 0.198) (Figure 6A). Sensitivity analyses revealed no evidence of horizontal pleiotropy (Egger intercept = −0.1055, *p* = 0.3271) or heterogeneity (Q = 1.7974 and *p* = 0.6155), and Leave-One-Out (LOO) analysis confirmed the robustness of the results (Table 3; Figure S10A). Similarly, urinary albumin-to-creatinine ratio (UACR) was not causally associated with PAD. In contrast, reverse-direction MR analysis identified a significant negative causal relationship between PAD and eGFR (OR = 0.997; 95% CI: 0.995–0.998; and adjusted *p* = 1.75 × 10^−3^) (Figure 6B**)**. No horizontal pleiotropy was detected (Egger intercept = −0.0003, *p* = 0.5639), and LOO analysis confirmed result robustness. However, significant heterogeneity was observed (Q = 32.7154, *p* = 0.0081) (Table 4; Figure S10B). Interestingly, PAD showed a marginally positive causal association with UACR (OR = 0.988; 95% CI: 0.978–0.998), although this association lost statistical significance after Benjamini-Hochberg correction (adjusted *p* = 0.051). No pleiotropy (Egger intercept = 0.0003, *p* = 0.8932) or heterogeneity (Q = 20.0755, *p* = 0.2168) was detected, and LOO analysis again confirmed the robustness of the results (Table 3; Figure S10C). Detailed information regarding the selected SNPs for MR analysis is provided in Tables S9–S11. In addition, MR analyses examining the effect of kidney function on pulse wave velocity (PWV) yielded positive causations, and sensitivity analyses demonstrated the robustness of these findings (Table S12).

**Table 4:** Sensitivity analyses for horizontal pleiotropy and heterogeneity in Mendelian randomization evaluating the causal effects between kidney function and PAD

Exposure	Outcome	Egger_intercept	SE_intercept	pval_intercept	Q	Q_df	Q_pval
eGFR	PAD	−0.1055	0.0820	0.3271	1.7974	3	0.6155
PAD	eGFR	−0.0003	0.0006	0.5639	32.7154	16	0.0081
PAD	UACR	0.0003	0.0025	0.8932	20.0755	16	0.2168

Sensitivity analyses were conducted to validate the robustness of the causal inference in the bidirectional Mendelian randomization (MR) models. Potential horizontal pleiotropy was evaluated using the MR-Egger regression intercept test, where a *p* value (pval_intercept) ≥ 0.05 indicates the absence of significant directional pleiotropic bias. Heterogeneity among the selected instrumental variables (SNPs) was quantified using Cochran’s Q statistic, with statistical significance (Q_pval) determined based on the corresponding degrees of freedom (Q_df).

PAD, peripheral artery disease; eGFR, estimated glomerular filtration rate; UACR, urinary albumin-to-creatinine ratio; SNP, single nucleotide polymorphism; Q, Cochran’s Q statistic; Q_df, degrees of freedom for Q; Q_pval, *p* value of Cochran’s *Q* test.

## Discussion

This study integrated evidence from global epidemiology, national health statistics, real-world clinical cohorts, and MR to better characterize the influence of kidney dysfunction on PAD. Although previous studies have shown that CKD worsens the prognosis of patients with PAD, our findings extend existing knowledge by demonstrating that this association is highly consistent across multiple independent data sources and by clarifying the possible direction of effect between PAD and subsequent decline in kidney function. Data from the GBD 2021 study indicated that kidney dysfunction has been a major and relatively stable contributor to PAD-related mortality worldwide. This pattern was also observed at the national level using the Centers for Disease Control and Prevention Wide-ranging Online Data for Epidemiologic Research (CDC WONDER) database. Analyses based on individual patient data from MIMIC-IV and a prospective cohort from China further confirmed that AKI/CKD markedly worsen clinical outcomes in patients with PAD. In addition, the MR analyses suggested a potential causal effect of PAD on reduced eGFR, whereas the opposite causal pathway was not supported. These results imply that genetic susceptibility to PAD may contribute to renal impairment through mechanisms not fully captured by traditional confounding factors. Taken together, the converging evidence from different levels of analysis highlights a bidirectional but asymmetric relationship between PAD and kidney dysfunction and underscores the need for more proactive assessment and protection of kidney function in patients with PAD.

Epidemiological studies have consistently shown that PAD is highly prevalent among patients with CKD and is associated with adverse clinical outcomes.^23^^,^^24^^,^^25^^,^^26^ Compared with non-diabetic individuals, the prevalence of PAD is approximately 3-fold higher in patients with diabetes. Furthermore, individuals with an eGFR <60 mL/min/1.73 m^2^ have a 6.5-fold increased risk of PAD compared with those with eGFR ≥60 mL/min/1.73 m^2^.^27^^,^^28^^,^^29^ The risk of developing PAD increases progressively with the severity of CKD. Importantly, patients with CKD undergoing PAD-related interventions such as revascularization or amputation are at significantly higher risk of poor outcomes and mortality.^27^^,^^30^^,^^31^^,^^32^ These findings have led to a growing clinical consensus that vascular complications should be a major focus in the management of patients with CKD.^33^

Similarly, accumulating evidence suggests that PAD may contribute to an increased risk of kidney function decline.^34^^,^^35^^,^^36^ Although some studies have reported no significant association between abnormal ankle-brachial index (ABI) and renal outcomes, recent findings have refined this view.^35^^,^^37^ In a prospective study of patients with type 2 diabetes, continuous monitoring of ABI revealed a reverse J-shaped association between mean ABI and CKD progression, whereby both abnormally low and high ABI values, as well as increases or decreases in annual mean ABI, were associated with a higher risk of CKD progression.^34^ Additional studies have supported PAD as a risk factor for CKD progression.^36^^,^^38^^,^^39^ Moreover, PAD has also been linked to a higher risk of AKI, particularly in the context of surgical procedures and contrast agent exposure.^40^^,^^41^^,^^42^ In our study of 2,133 PAD patients, 32.30% had comorbid CKD, further highlighting the high prevalence and potential risk of kidney dysfunction in individuals with PAD.

In patients with PAD, the presence of CKD is associated with a markedly increased risk of adverse outcomes.^23^^,^^43^^,^^44^ In a large retrospective analysis involving 874,788 PAD patients, 12.2% had comorbid CKD, and the rate of limb amputation increased significantly with advancing CKD stage.^22^ Among patients with both CKD stage 5 and diabetes, the amputation rate reached 36.5%. Compared to PAD patients without CKD, those with CKD stage 5 had a 4-fold higher risk of amputation and a 5-fold higher risk of mortality. These findings underscore the critical importance of CKD management in PAD patients to mitigate the risk of limb loss and death.

Furthermore, our study highlights a significant association between kidney dysfunction (CKD and AKI) and increased mortality in PAD patients. Importantly, at the global level, kidney dysfunction emerges as a major and consistently elevated risk factor for PAD-related death. Its remarkably stable and persistently high burden underscores a critical gap in the current management of PAD patients: insufficient attention to kidney function.

Notably, the bidirectional MR analysis revealed a causal association between genetic liability to PAD and lower eGFR, whereas no genetic effect of impaired kidney function on PAD was observed. This asymmetry reflects the fundamental difference between inherited susceptibility and acquired disease progression. MR captures lifelong genetic exposure,^45^ while the interaction between PAD and kidney dysfunction in clinical settings is largely shaped by postnatal factors—such as aging, inflammation, hemodynamic alterations, acute ischemic insults, and medication constraints, which are not determined by germline variation and therefore cannot be detected through MR analysis. The modest genetic effect of PAD on eGFR is biologically plausible, as the genetic instruments predominantly represent atherosclerotic susceptibility rather than anatomical disease burden or acute renal hypoperfusion capable of directly reducing kidney function. In contrast, the adverse impact of kidney dysfunction on PAD outcomes documented in clinical studies is driven by acquired pathological processes, including anemia, mineral metabolism disorders, and uremia-related endothelial injury, which are not genetically mediated. Thus, the genetic causal direction suggested by MR does not contradict clinical evidence but complements it by addressing a different layer of disease mechanisms. Taken together, findings across multiple data sources consistently underscore the central role of kidney dysfunction in the progression and prognosis of PAD.

PWV is considered the gold standard for assessing arterial stiffness, and multiple large-scale cohort studies have demonstrated that higher PWV values are significantly associated with an increased risk of PAD.^46^ Moreover, among patients with established PAD, PWV reflects not only arterial stiffness but also is directly associated with functional capacity, with higher brachial-ankle PWV (baPWV) associated with shorter walking distances during exercise testing.^47^ In this study, in the absence of preferred clinical indicators such as the ABI, we additionally incorporated data from three PWV measures and attempted to use these metrics as proxies to simulate different degrees of PAD severity, in order to evaluate the potential genetic association between kidney function indicators and arterial stiffness.^48^ However, there existed no causation between eGFR/UACR and PWV. These findings suggest that the role of kidney dysfunction in the development and progression of PAD may be better explained by phenotypic coexistence and cumulative risk burden, rather than through a direct genetic causal pathway or an arterial stiffness–mediated mechanism.

In fact, growing evidence indicates that the association between PAD and kidney dysfunction extends beyond epidemiological correlation and involves multiple overlapping vascular and metabolic pathways. PAD is increasingly recognized as a systemic manifestation of atherosclerosis rather than a disease confined to the lower extremities.^49^ Reduced arterial compliance, increased diffuse plaque burden, and endothelial dysfunction may impair renal perfusion reserve, particularly under conditions of hemodynamic instability or increased metabolic demand.^50^ Chronic limb ischemia has also been associated with activation of the renin-angiotensin-aldosterone system and heightened sympathetic tone, which may contribute to glomerular hypertension, proteinuria, and accelerated decline in eGFR.^51^ In a prospective multicenter cohort of 897 patients, a lower ankle-brachial index was independently associated with a faster annual decline in eGFR,^50^ suggesting that systemic vascular alterations related to PAD may influence kidney function progression.

At the microvascular level, PAD and CKD share several pathological features. Prior studies have demonstrated capillary rarefaction, reduced endothelial nitric oxide bioavailability, and impaired angiogenic signaling in skeletal muscle from patients with PAD and in renal tissue from individuals with CKD.^52^^,^^53^ The coexistence of macrovascular disease and microvascular dysfunction may further limit effective renal perfusion, exacerbate local hypoxia, and promote disease progression.^54^ In addition, CKD is characterized by elevated circulating uremic toxins, such as indoxyl sulfate, which have been shown to promote vascular smooth muscle proliferation and endothelial injury.^55^^,^^56^ PAD has likewise been linked to heightened systemic inflammation, including increased levels of interleukin 6, tumor necrosis factor-α, and activation of the NLRP3 inflammasome pathway.^57^ These inflammatory mediators are known to directly impair glomerular endothelial integrity and podocyte stability.^58^

At the metabolic level, mitochondrial dysfunction has been proposed as a shared molecular substrate linking PAD and kidney dysfunction. Experimental and clinical studies indicate that PAD-related skeletal muscle degeneration and renal tubular vulnerability in CKD are both accompanied by impaired mitochondrial oxidative phosphorylation and increased reactive oxygen species production.^59^^,^^60^ Multi-omics analyses have further identified concordant abnormalities in tricarboxylic acid cycle activity and lipid metabolism pathways in patients with PAD and CKD.^61^^,^^62^ These metabolic alterations may reduce tissue tolerance to ischemia-reperfusion stress and have been implicated in susceptibility to AKI.^63^ However, these lines of evidence do not establish a causal relationship between PAD and kidney dysfunction, and further experimental and longitudinal studies are required for validation.

Collectively, our findings provide robust evidence to support the integration of kidney function assessment into the specialized management of PAD, particularly in vascular surgery and related disciplines, emphasizing the need for a more holistic, cardiorenal approach to care. Collectively, our findings suggest that early screening for CKD using eGFR and albuminuria in patients with PAD, combined with vascular-renal co-management, may help refine risk stratification and inform integrated therapeutic strategies.

In clinical practice, PAD patients with reduced eGFR and/or albuminuria may benefit from early nephrology referral to optimize cardiovascular-renal risk stratification, guide medication selection, and inform peri-procedural management. A multidisciplinary vascular-renal care model, particularly for patients with advanced PAD or undergoing revascularization, may help improve long-term outcomes through coordinated risk assessment and longitudinal follow-up.

### Limitations of the study

Of course, our study has several limitations. First, in the MIMIC-IV database analysis, kidney disease diagnoses were recorded at the time of hospital admission for patients with PAD patients; therefore, the actual kidney function status at 30-, 180-, and 365-day post-admission could not be clearly determined. Moreover, MIMIC-IV primarily includes hospitalized and critically ill patients, and lacks systematic PAD severity measures (e.g., ABI or imaging-based grading), as well as detailed information on frailty, nutritional status, and outpatient medication use, which may limit representativeness and introduce residual confounding. Second, the GBD database analysis relies on modeling strategies defined by the GBD consortium, which may involve residual confounding that we are unable to fully address. Third, in the MR analysis, while we identified a causal effect of PAD on decreased eGFR from a genetic perspective, the lack of a causal effect of eGFR on PAD was observed within the specific context and assumptions of this analytical framework. Additionally, due to the limited number of SNPs reaching genome-wide significance for certain traits, a relaxed threshold *p* < 5e^−6^ was used to ensure a sufficient number of instruments for MR analysis. Although we ensured instrument strength via F-statistics, this approach might still affect the robustness of the causal inferences. In addition, despite the integration of multiple large public datasets, the retrospective design and reliance on estimated GFR, which is widely used and guideline-recommended, could limit mechanistic inference. Finally, although this study integrates evidence from multiple large public databases and diverse populations, its retrospective design and reliance on genetic epidemiological analyses limit mechanistic inference. Large database-based analyses are also subject to inherent limitations related to data availability and population representativeness. Future studies incorporating multi-omics approaches and refined phenotyping are warranted to validate these associations and clarify potential causal pathways.

## Resource availability

### Lead contact

For additional information or requests for resources and reagents, please contact the lead contact, Guoping Zheng (guoping.zheng@sydney.edu.au).

### Materials availability

No new unique reagents or materials were generated in this study.

### Data and code availability

• All publicly available datasets analyzed in this study were obtained from the following repositories: MIMIC-IV (https://physionet.org/content/mimiciv/), GBD (https://vizhub.healthdata.org/gbd-results/), CDC WONDER (https://wonder.cdc.gov/), CKDGen (http://ckdgen.imbi.uni-freiburg.de/), and FinnGen (https://www.finngen.fi/en/access_results).
• The code supporting the analyses in this study has been uploaded to GitHub: https://github.com/HW-Eternity/PAD_Raw-Code.git.
• The original clinical data for the 2H-SXMU cohort were obtained from the participating hospital and are available from the lead contact upon reasonable request.
• Any additional information required to reanalyze the data reported in this paper is available from the lead contact upon request.

## Acknowledgments

This work was supported by the 10.13039/501100000925National Health and Medical Research Council (NHMRC) of Australia (grant no. 2027965). The authors extend their gratitude to the participants of the MIMIC-Ⅳ database, GBD database, CKDGen consortium, and FinnGen database for their invaluable contributions.

## Author contributions

Study conception and design, H.W., C.F., K.F., and G.Z.; data collection, H.W., C.F., K.F., Y.N., Z.W., Y.L., R.C., J.J., J.H., Y.S., Y.W., S.W., and K.X.; data analysis and interpretation, H.W., C.F., K.F., Y.N., Z.W., Y.L., and Y.S.; statistical analysis, H.W., C.F., K.F., Y.N., Z.W., R.C., Y.W., S.W., and K.X.; supervision, G.Z. and Y.Z. All authors contributed substantially to the drafting or critical revision of the manuscript.

## Declaration of interests

The authors declare no conflicts of interest.

## STAR★Methods

### Key resources table

**Table:** 

REAGENT or RESOURCE	SOURCE	IDENTIFIER
**Deposited data**		

Original code for data analysis	https://github.com/HW-Eternity/PAD_Raw-Code.git	N/A
MIMIC-IV database	https://physionet.org/content/mimiciv/	N/A
Global Burden of Disease	https://vizhub.healthdata.org/gbd-results/	N/A
CDC WONDER database	https://wonder.cdc.gov/	N/A
CKDGen consortium data	http://ckdgen.imbi.uni-freiburg.de/	N/A
FinnGen results	https://www.finngen.fi/en/access_results	N/A

**Software and algorithms**		

R	The R Project for Statistical Computing	Version 4.2.2
GraphPad	GraphPad Software	Version 10.4.0
SPSS	SPSS Software	Version 26.0

### Experimental model and study participant details

#### Human participant study

The 2H-SXMU study was approved by the Shanxi Medical University Second Hospital Medical Research Ethics Committee (Approval No. [2024]YX211).

Inclusion criteria were: (1) age between 18 and 85 years; (2) diagnosis of PAD; and (3) first-time endovascular treatment involving balloon angioplasty with stent implantation of the superficial femoral artery.

Exclusion criteria were: (1) non-atherosclerotic causes of lower-extremity arterial stenosis (e.g., vasculitis); (2) target lesion not located in the superficial femoral artery; (3) endovascular procedures other than balloon angioplasty with stent implantation; and (4) history of prior lower-extremity revascularization.

A total of 200 patients with PAD who were treated in the Department of Vascular Surgery in the Second Hospital of Shanxi Medical University (2H-SXMU) between January and May 2024 were consecutively enrolled. All included patients underwent their first PAD-related endovascular procedure, consisting of balloon angioplasty followed by stent implantation of the superficial femoral artery (SFA).

### Method details

#### GBD database study

The GBD study, coordinated by the Institute for Health Metrics and Evaluation (IHME), is a comprehensive and systematic effort to quantify the impact of diseases, injuries, and risk factors worldwide.^64^ The GBD 2021 study provides comprehensive estimates of the incidence, mortality, and burden of diseases and injuries across 204 countries, 21 GBD regions, and 5 socio-demographic index (SDI) levels from 1990 to 2021.

Disability-Adjusted Life Years (DALYs) were calculated as the sum of Years of Life Lost due to premature mortality and Years Lived with Disability. To eliminate the effect of differences in population age structures, all rates were age-standardized using the GBD world standard population.^65^ We retrieved data on PAD-specific age-standardized prevalence rate (ASPR), age-standardized death rate (ASDR), and age-standardized disability-adjusted life year rate (ASDALYR) per 100,000 population. SDI, a composite measure of income per capita, average educational attainment, and total fertility rate, was used to evaluate the development status of regions. Population-attributable fractions (PAF) for key risk factors were also obtained to quantify the proportion of PAD burden (deaths and DALYs) that could be reduced if the exposure to certain risk factors (e.g., smoking, high blood pressure) were reduced to the theoretical minimum risk exposure level.^64^

#### CDC WONDER database study

We investigated national trends in mortality associated with PAD combined with CKD and PAD combined with AKI in the United States from 1999 to 2023. Mortality data were provided by the National Center for Health Statistics (NCHS) and obtained through the Centers for Disease Control and Prevention Wide-ranging Online Data for Epidemiologic Research (CDC WONDER), which compiles death certificate records from all 50 U.S. states and the District of Columbia.

According to the International Classification of Diseases, Tenth Revision (ICD-10), CKD was identified using N18 (N18.0–N18.9). AKI were identified using N17 (N17.0–N17.9). PAD was identified using I73.0, I73.1, I73.8, and I73.9.

The study included individuals aged ≥25 years, grouped in ten-year intervals from 1999 to 2023. This study followed the STROBE guidelines.^66^ Because the study used publicly available, de-identified, and anonymized government data, institutional review board approval was not required.

In addition, demographic characteristics and geographic region of residence were extracted for the study population. Stratified analyses were conducted according to sex, age group, race and ethnicity as defined by the U.S. Census Bureau (Hispanic or Latino; non-Hispanic Black or African American [NH-Black]; non-Hispanic White [NH-White]; and non-Hispanic other races [NH-Other]), and census region (Northeast, Midwest, South, and West).^67^ Age-adjusted mortality rates (AAMRs) were calculated using the direct standardization method with the 2000 U.S. standard population, in order to reduce bias due to temporal changes and shifts in population structure.^68^

#### MIMIC-IV database study

The MIMIC-IV database is a publicly available, comprehensive critical care dataset containing de-identified clinical information on ICU admissions at Beth Israel Deaconess Medical Center from 2008 to 2019. It includes detailed data on demographics, laboratory results, medications, vital signs, and diagnostic codes. It serves as a valuable resource for research in critical care, epidemiology, and health outcomes. For the present study, C. F. completed the required human subjects training (Certification ID: 64447378) and was responsible for data extraction and preprocessing in accordance with ethical standards and MIMIC-IV data use guidelines.

PAD was defined as atherosclerotic narrowing or occlusion of the limb arteries, typically presenting as intermittent claudication, rest pain, or limb ulcers. Diagnosis was based on the first hospitalization with relevant ICD codes (ICD-10: I70.2–I70.8, I73–I73.9; ICD-9: 440.2, 440.4, 443.0–443.9), confirmed by clinical and/or imaging findings. CKD was defined as a documented diagnosis of CKD or hospital records consistent with CKD, determined from clinical notes, laboratory values, or diagnosis codes. Exclusion criteria included: (1) length of stay <48 hours; (2) age <18 years; (3) active or prior malignancy; (4) missing critical data. Outcomes were compared between patients with PAD with versus without CKD.

Data were extracted from the PostgreSQL-based relational structure of MIMIC-IV. Only the first hospital admissions were included to ensure consistency. The variables were selected based on their clinical significance in predicting outcomes for patients with PAD and kidney dysfunction, as well as their common use in ICU risk stratification.^69^ Variables collected included demographics (age, sex, race), which are standard for baseline adjustment in large-scale database studies.^70^ Vital signs reflecting hemodynamic status and acute physiological stress were recorded: heart rate, SpO_2_, respiratory rate (RR), systolic (SBP), diastolic (DBP), and mean arterial pressure (MAP),^71^ To account for systemic disease burden and potential confounding factors, we included clinical conditions: hypertension,^72^ diabetes, heart failure,^72^ atherosclerosis, AKI, CKD.^4^Treatments representing life-support intensity, such as mechanical ventilation (MV),^73^ continuous renal replacement therapy (CRRT)^74^ were also extracted. Laboratory values providing quantitative evidence of metabolic and renal status included glucose,^75^ SCr, blood urea nitrogen (BUN),^1^ urine output, red blood cell count, white blood cell count, and platelet count.^76^ Finally, the Severity of illness was assessed using the Sequential Organ Failure Assessment (SOFA) score was utilized as a composite measure of acute organ dysfunction severity upon admission.^69^ The primary outcomes were all-cause mortality at 30-, 180-, and 365-days post-admission.

Patients with missing key indicators at baseline were excluded as per the predefined criteria (Figure 1). Patients with PAD and CKD comprised the exposure group; those without CKD served as controls. The normality of continuous variables was assessed using the Kolmogorov–Smirnov test. Besides, time-varying Cox regression models were constructed by segments (Day 30, 180, and 365). Kaplan–Meier survival curves were generated for mortality at 30, 180, and 365 days. Subgroup analyses were conducted using Cox proportional hazards models to evaluate the association between CKD and mortality (at 180 and 365 days) across strata defined by sex, race, and comorbidities (AKI, heart failure, atherosclerosis, diabetes, and hypertension).

#### Clinical cohort study of 2H-SXMU

A total of 200 patients with PAD who were treated in the Department of Vascular Surgery in the Second Hospital of Shanxi Medical University (2H-SXMU) between January and May 2024 were consecutively enrolled. All included patients underwent their first PAD-related endovascular procedure, consisting of balloon angioplasty followed by stent implantation of the superficial femoral artery (SFA).

All patients were followed for 12 months after the index procedure. The primary outcome was the occurrence of major adverse limb events (MALE) within one year after surgery. MALE was defined as major amputation or repeat lower-extremity revascularization, including endovascular or surgical reintervention.^21^ Inclusion criteria were: (1) age between 18 and 85 years; (2) diagnosis of PAD; and (3) first-time endovascular treatment involving balloon angioplasty with stent implantation of the superficial femoral artery. Exclusion criteria were: (1) non-atherosclerotic causes of lower-extremity arterial stenosis (e.g., vasculitis); (2) target lesion not located in the superficial femoral artery; (3) endovascular procedures other than balloon angioplasty with stent implantation; and (4) history of prior lower-extremity revascularization.

#### MR study

MR is a genetic epidemiological method that uses single nucleotide polymorphisms (SNP) as instrumental variables (IVs) to infer causal relationships between exposures and outcomes. In this study, bidirectional MR was applied to evaluate the potential causal link between kidney dysfunction and PAD.

GWAS summary statistics for kidney function were obtained from the CKDGen consortium (https://ckdgen.imbi.uni-freiburg.de/), including estimated glomerular filtration rate (eGFR) data from 567,460 European participants and UACR data from 348,954 European participants.^77^ GWAS data for PAD were derived from FinnGen (https://www.finngen.fi/, finn-b-I9_PAD), including 7,098 cases and 206,541 controls of European ancestry. GWAS data for pulse wave velocity (PWV) were obtained from the Leipzig Health Atlas database (https://www.health-atlas.de/studies/40), including carotid–femoral PWV (cfPWV), brachial–ankle PWV (baPWV), and brachial–femoral PWV (bfPWV). These data were derived from a randomly sampled, population-based study comprising 7,669 participants from the LIFE-Adult cohort and 532,676 SNPs.^48^ (Table S1).

To ensure the validity of the causal inference, our MR study strictly adhered to the three fundamental assumptions.^78^^,^^79^ (1) Relevance assumption: The instrumental variables (IVs) must be strongly associated with the exposure. To verify this and avoid weak instrument bias, SNPs with r^2^ < 0.001, P < 5×10^-6^, F-statistics > 10 and a minor allele frequency ≥ 0.01 was selected. Ambiguous or palindromic SNPs were also excluded to ensure strand alignment. (2) Independence assumption: The IVs must be independent of any confounders that might influence the exposure-outcome relationship. To satisfy this, SNPs associated with potential confounders (e.g., smoking, alcohol intake, BMI) were systematically identified and excluded using the LDtrait tool. (3) Exclusion restriction assumption: The IVs must influence the outcome exclusively through the exposure, without horizontal pleiotropy. This assumption was verified using multiple sensitivity analyses. Specifically, the MR-Egger intercept test was employed to detect horizontal pleiotropy, and Cochran’s Q statistic was used to assess heterogeneity. Furthermore, leave-one-out analysis was conducted to assess whether the causal estimates were driven by any single potentially pleiotropic SNP. Besides, five MR methods were applied: inverse-variance weighted (IVW), weighted median estimator (WME), MR-Egger regression (MR-ER), simple mode, and weighted mode.

### Quantification and statistical analysis

Statistical analyses were performed using R software (version 4.2.2), GraphPad Prism (version 10.4.0), and SPSS (version 26.0). In all relevant figures, statistical significance is indicated by asterisks (e.g., ∗, p < 0.05; ∗∗, p < 0.01; ∗∗∗, p < 0.001), which are defined in the corresponding figure legends along with the specific statistical tests used and the number of biological units (*n*). All detailed statistical parameters, including exact p values, degrees of freedom, and effect sizes (HR, OR, 95% CI/UI, and F-statistics), are provided either in the results or the figure legends. Data normality was assessed using the Kolmogorov–Smirnov test. Continuous variables are presented as mean ± SD for normal data or median with IQR for skewed data. Categorical variables are reported as counts and percentages. For GBD and CDC WONDER studies, age-standardized rates were compared using 95% UI or CI. For clinical cohorts (MIMIC-IV and 2H-SXMU), group comparisons were conducted using the independent-samples t test, Mann–Whitney U test, Pearson’s χ^2^ test, or Fisher’s exact test as appropriate.

Specifically, age-standardized rates (ASPR, ASDR, ASDALYR) from GBD and age-adjusted mortality rates (AAMRs) from CDC WONDER were compared using 95% uncertainty intervals (UI) or confidence intervals (CI).

For MIMIC-IV, all variables had <5% missingness, addressed using Multiple Imputation by Chained Equations (MICE) via the “mice” package in R to ensure data integrity and minimize selection bias. Since most clinical variables (e.g., SOFA scores and laboratory values) exhibited a skewed distribution, the non-parametric Mann–Whitney U test was employed for comparisons, and data were presented as medians with interquartile ranges (IQRs). Propensity score matching (PSM) was performed using a caliper width of 0.1 to ensure baseline covariate balance. To address the potential violation of the proportional hazards (PH) assumption over long-term follow-up. Survival analysis was conducted using Kaplan–Meier curves and time-varying Cox regression models to estimate hazard ratios (HR). The PH assumption for each segment was verified using Schoenfeld residuals tests (all p > 0.05). To assess the consistency of the association and identify potential effect modification, interaction tests were performed by including multiplicative interaction terms in the Cox models. The p-values for interaction were calculated using the likelihood ratio test.

For the MR study, the Inverse-Variance Weighted (IVW) method served as the primary analysis, supplemented by WME, MR-Egger, and MR-PRESSO. Horizontal pleiotropy and heterogeneity were assessed using the MR-Egger intercept and Cochran’s Q statistic. To control for the false discovery rate (FDR) from multiple comparisons, the Benjamini-Hochberg (BH) procedure was applied to the p-values of primary estimates. An adjusted P < 0.05 was considered statistically significant.
