# Supplementary material for: Impact of kidney dysfunction on outcomes in peripheral artery disease: A multi-database cohort study
Source: iScience. 2026 Apr 10;29(5):115681. doi: 10.1016/j.isci.2026.115681 (PMC13133972; doi:10.1016/j.isci.2026.115681)
Supplement: Document S1. Figures S1–S10 and Tables S1–S13 [file mmc1.pdf]

iScience, Volume *29*

Supplemental information

Impact of kidney dysfunction on outcomes

in peripheral artery disease:

A multi-database cohort study

Heng Wang, Chaonan Fan, Keyi Fan, Yijie Ning, Ziyan Wang, Yaling Li, Runze Chang, Jianhua Jiang, Jiang Han, Yongbin Shi, Yuwen Wang, Shule Wang, Yimiao Wei, Keyang Xu, Yun Zhou, and Guoping Zheng

Supplementary Figures

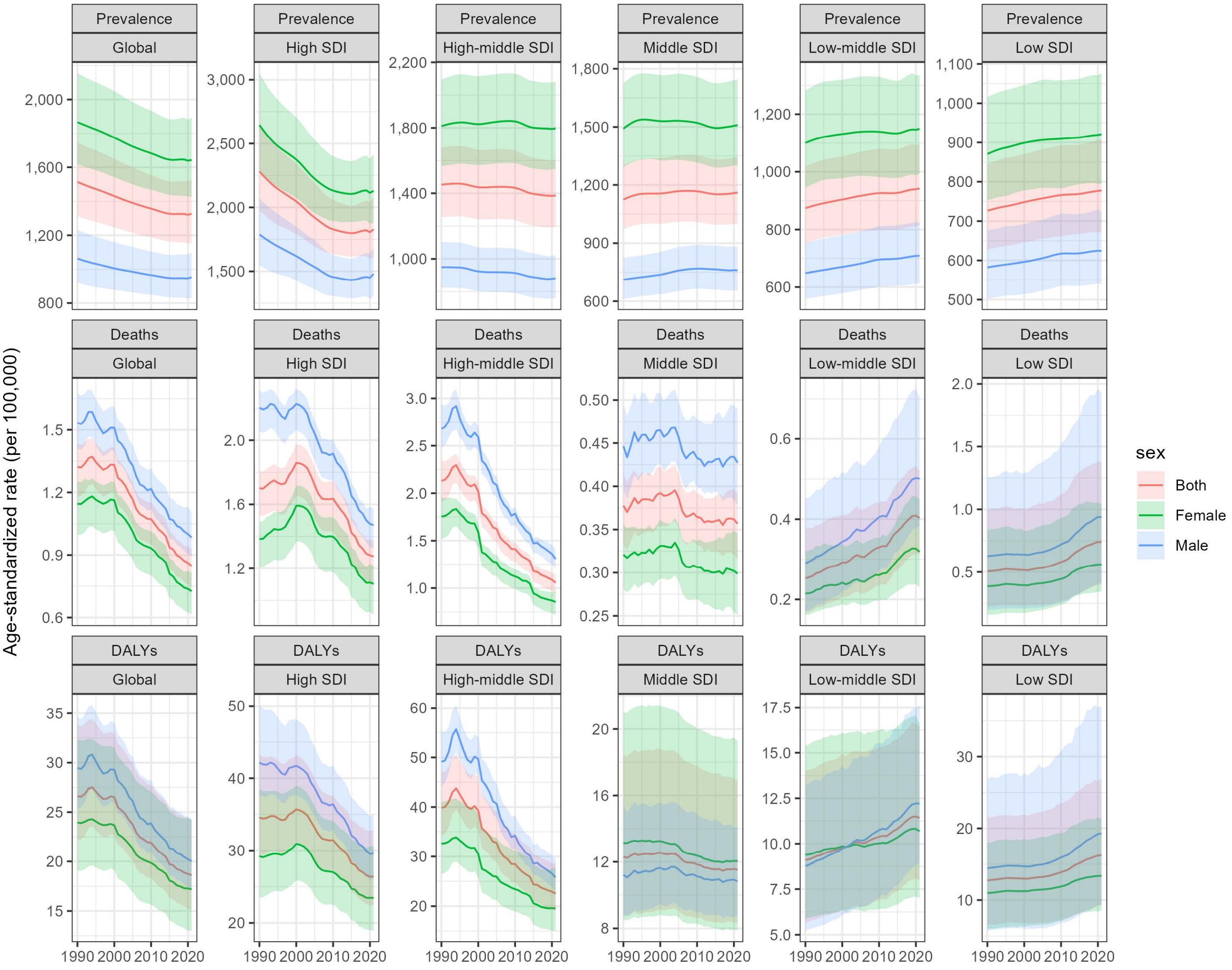

Figure S1: Trends in the global and regional burden of peripheral artery disease (PAD) from 1990 to 2021. Age-standardized prevalence rate (ASPR), age-standardized death rate (ASDR), and age-standardized disability-adjusted life years rate (ASDALYR) for PAD are shown globally and across the five sociodemographic index (SDI) regions.

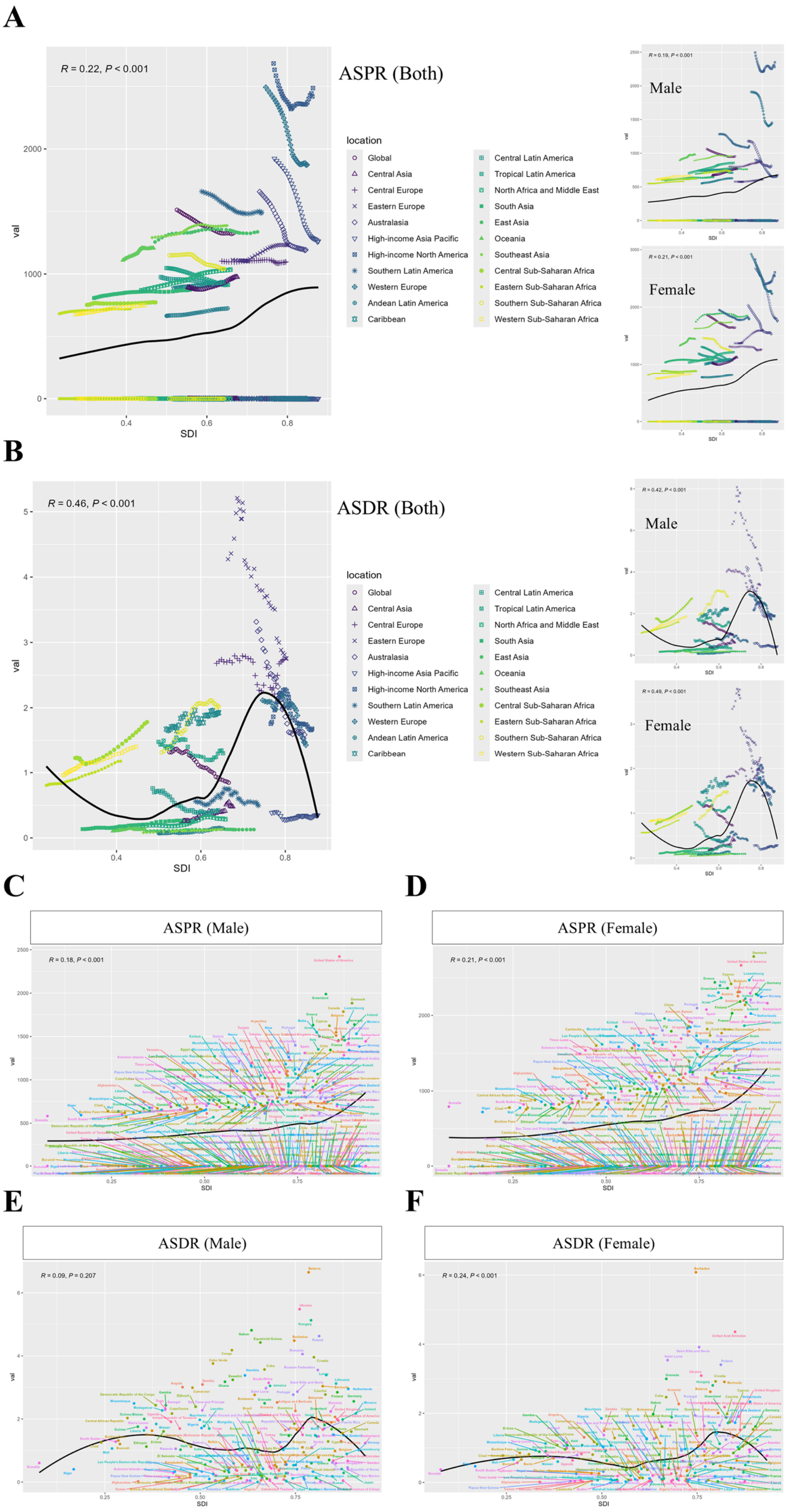

Figure S2: Correlation between sociodemographic index (SDI) and the burden of peripheral artery disease (PAD). (A) Correlation between age-standardized prevalence rate (ASPR) and SDI across 21 Global Burden of Disease (GBD) regions. **(B)** Correlation between age-standardized death rate (ASDR) and SDI across 21 GBD regions. **(C, D)** Correlation between SDI and ASPR across 204 countries and territories. **(E, F)** Correlation between SDI and ASDR across 204 countries and territories. Statistical significance was determined using Spearman's rank correlation analysis. The Spearman's correlation coefficient (*R*) and exact *p* values are indicated within each panel.

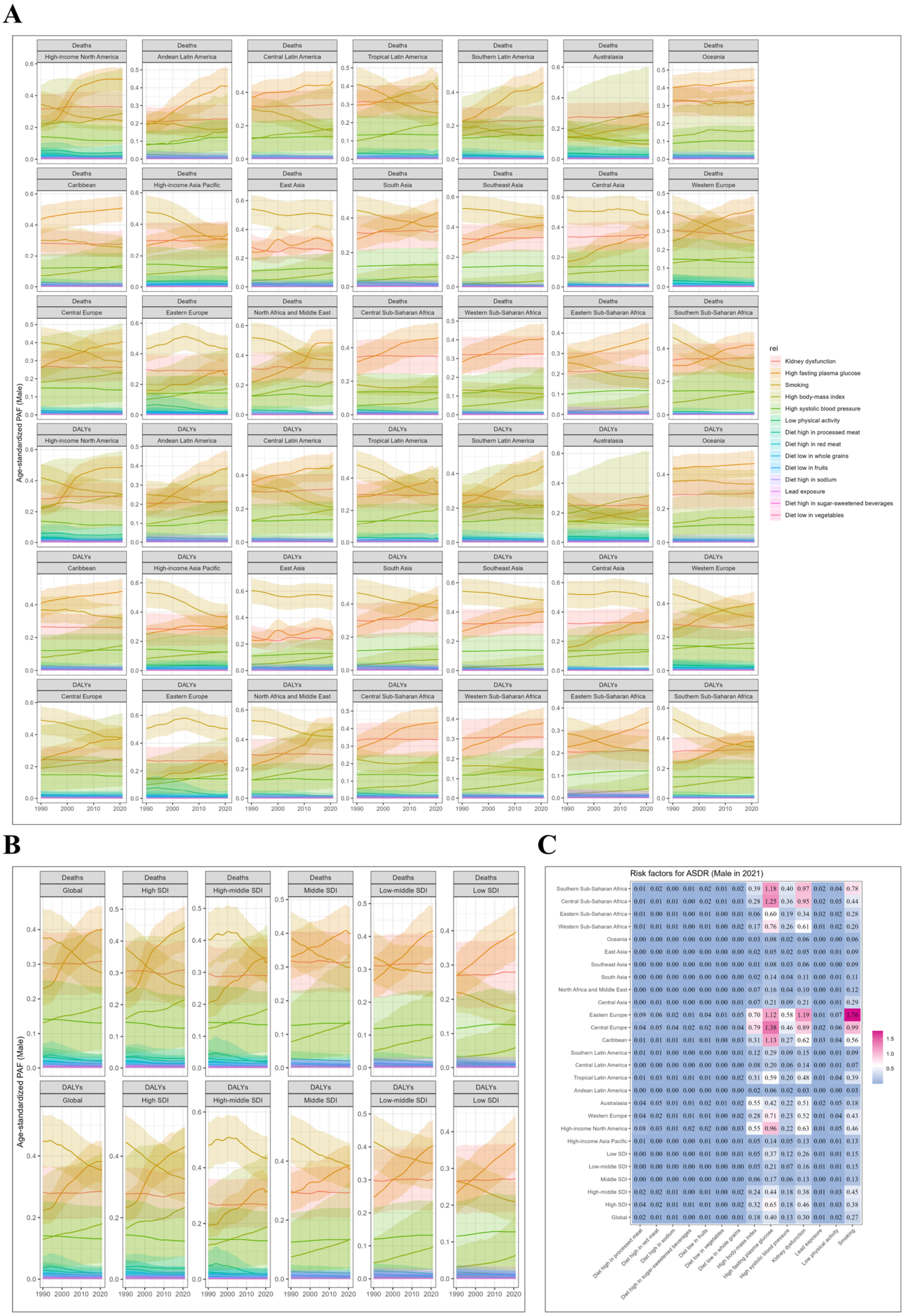

Figure S3: Global risk factors for peripheral artery disease (PAD) in male patients. (A) Population attributable fractions (PAF) of risk factors for PAD-related deaths in males across 21 Global Burden of Disease (GBD) regions from 1990 to 2021. **(B)** PAFs of PAD-related deaths in males globally and across five sociodemographic index (SDI) regions from 1990 to 2021. **(C)** Age-standardized death rates (ASDR) of PAD risk factors in males globally in 2021.

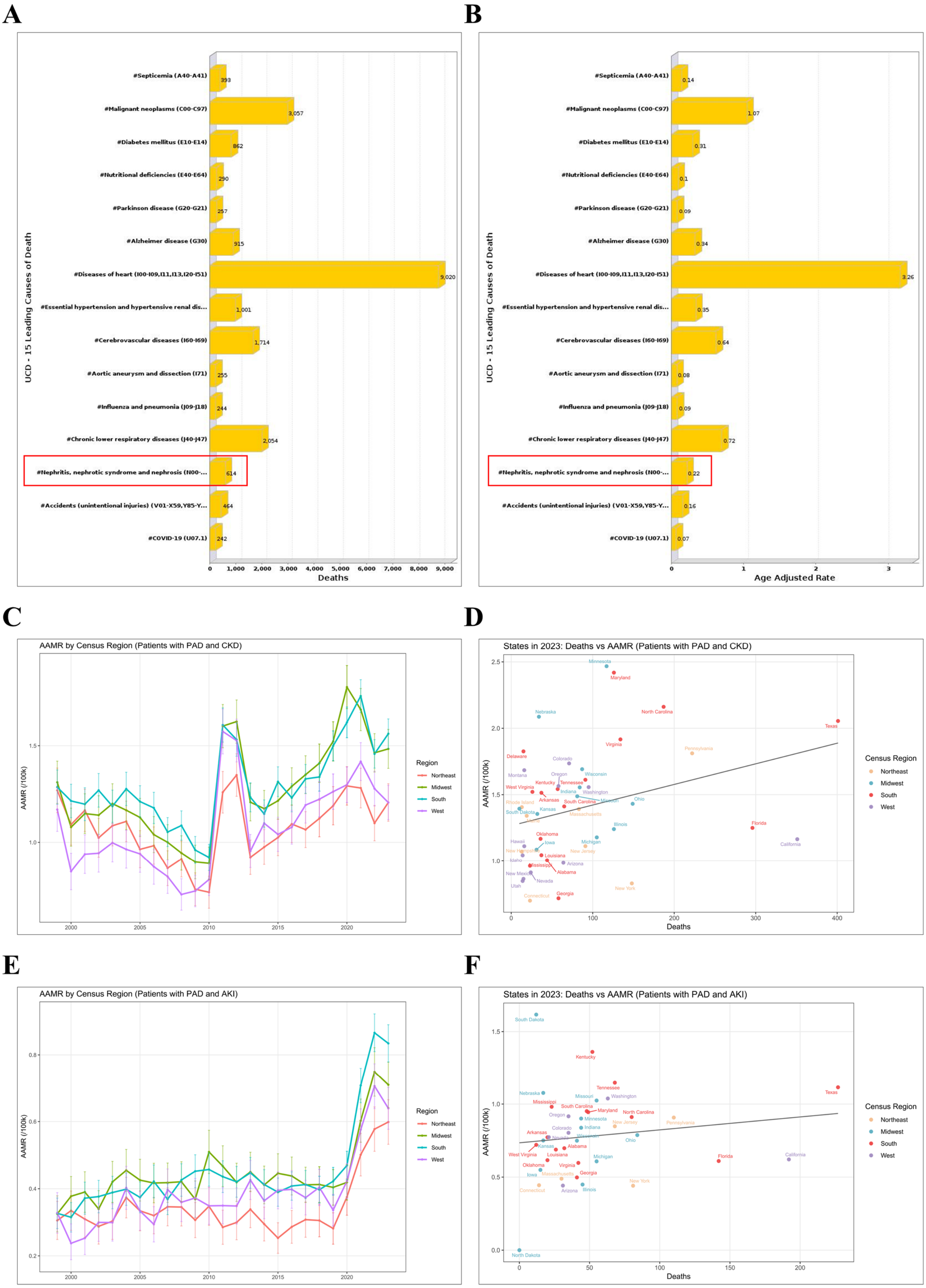

Figure S4. Mortality patterns among PAD decedents with kidney dysfunction in the United States.

(A–B) Leading causes of death among PAD decedents with kidney disease. **(C–F)** Temporal trends and geographic distribution of age-adjusted mortality rates for PAD combined with CKD or AKI across U.S. regions and states.

A

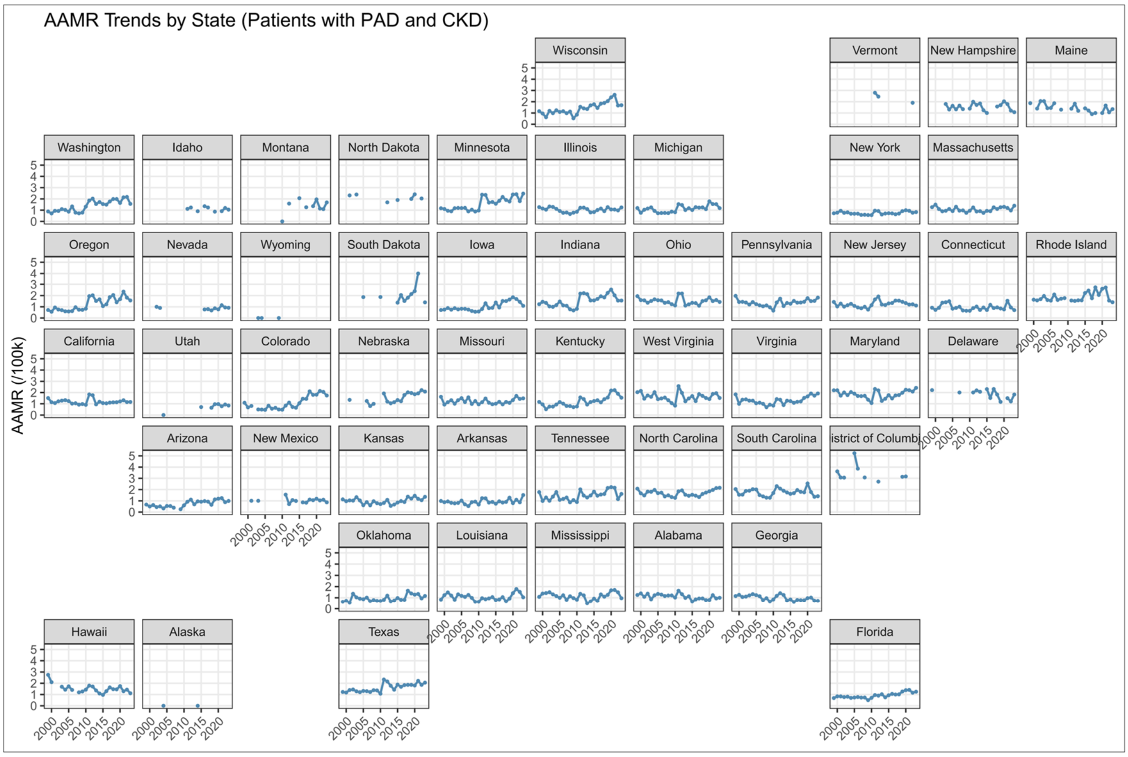

B

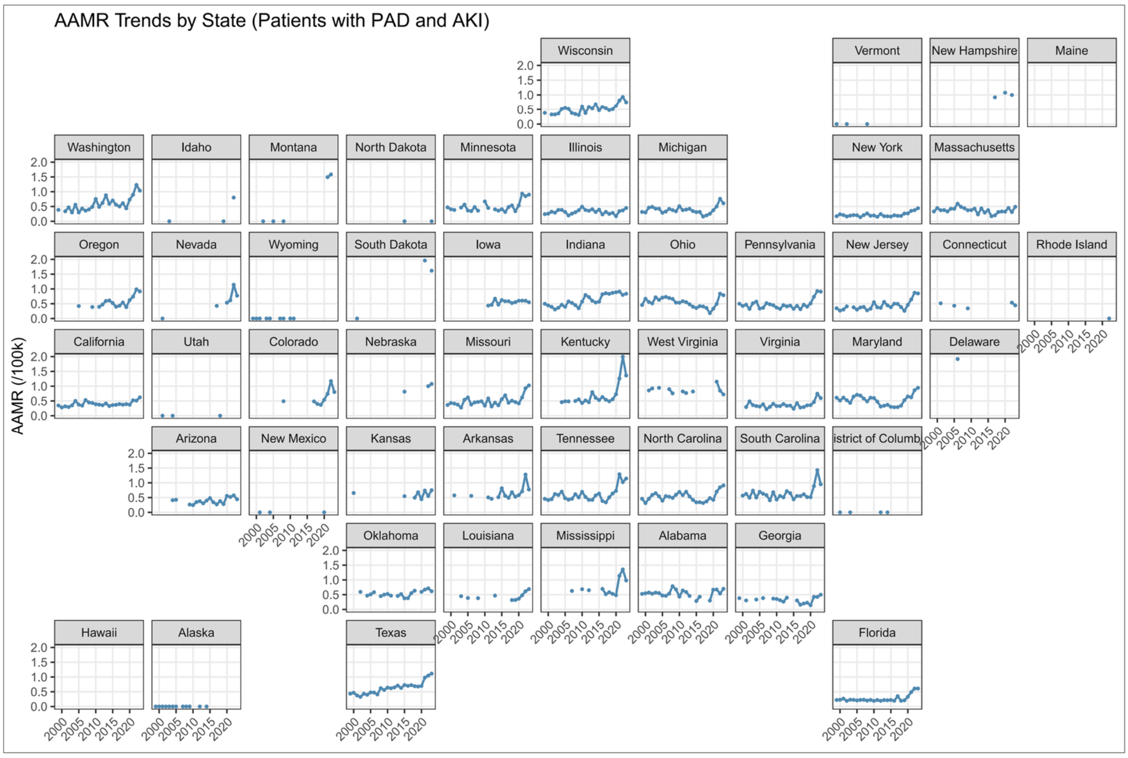

Figure S5. Age-adjusted mortality rates (AAMR) among PAD patients with kidney dysfunction across U.S. states, 1999–2023.

(A) Patients with PAD and CKD**;** **(B)** Patients with PAD and AKI.

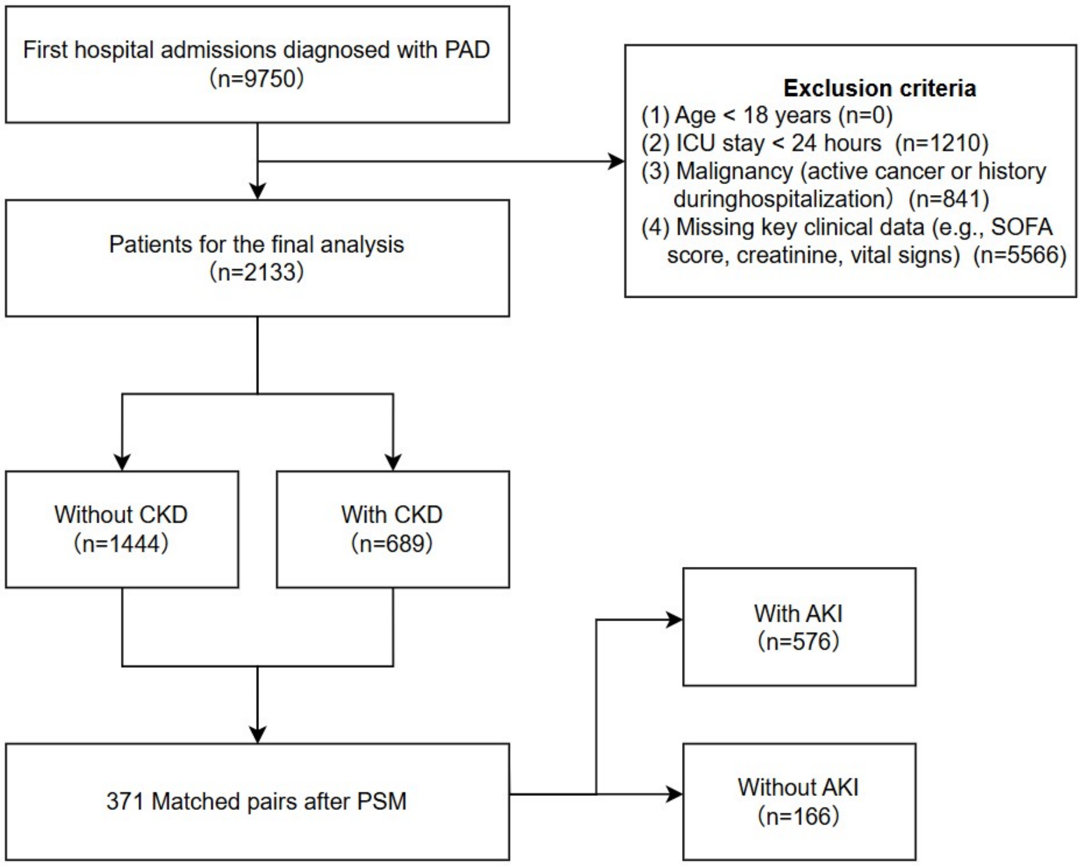

Figure S6: Flow chart of population selection.

Among 9,750 patients with a first hospital admission diagnosis of peripheral artery disease (PAD), 7,617 were excluded based on the following criteria: age < 18 years (n = 0), intensive care unit (ICU) stay < 24 hours (n = 1,210), malignancy (active or historical cancer during hospitalization; n = 841), and missing key clinical data (e.g., Sequential Organ Failure Assessment [SOFA] score, serum creatinine, or vital signs; n = 5,566). A total of 2,133 patients were included in the final analysis, including 1,444 patients without chronic kidney disease (CKD) and 689 patients with CKD. After propensity score matching (PSM), 371 matched pairs were obtained for subsequent analyses.

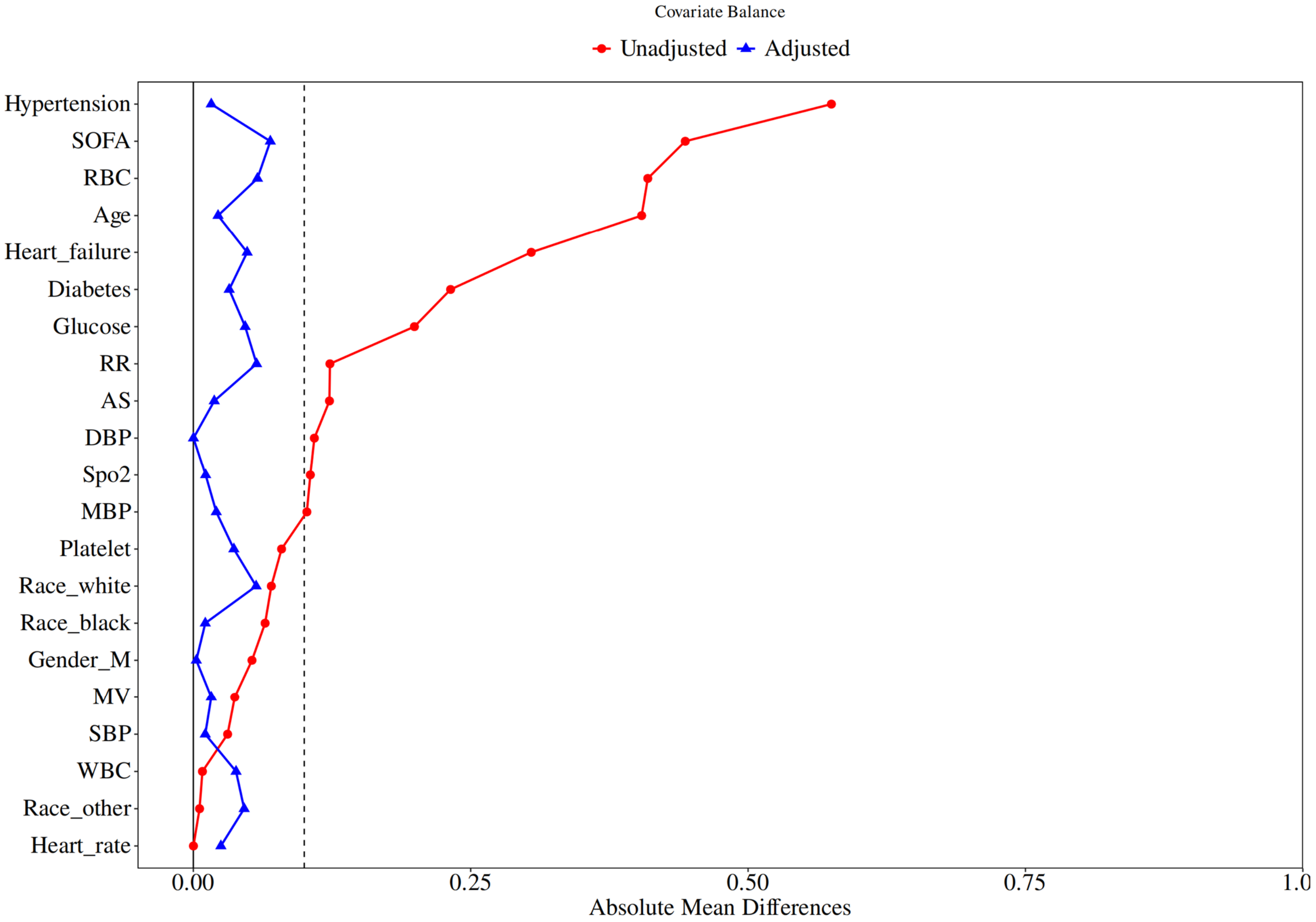

Figure S7: Standardized mean differences of baseline covariates before and after propensity score matching.

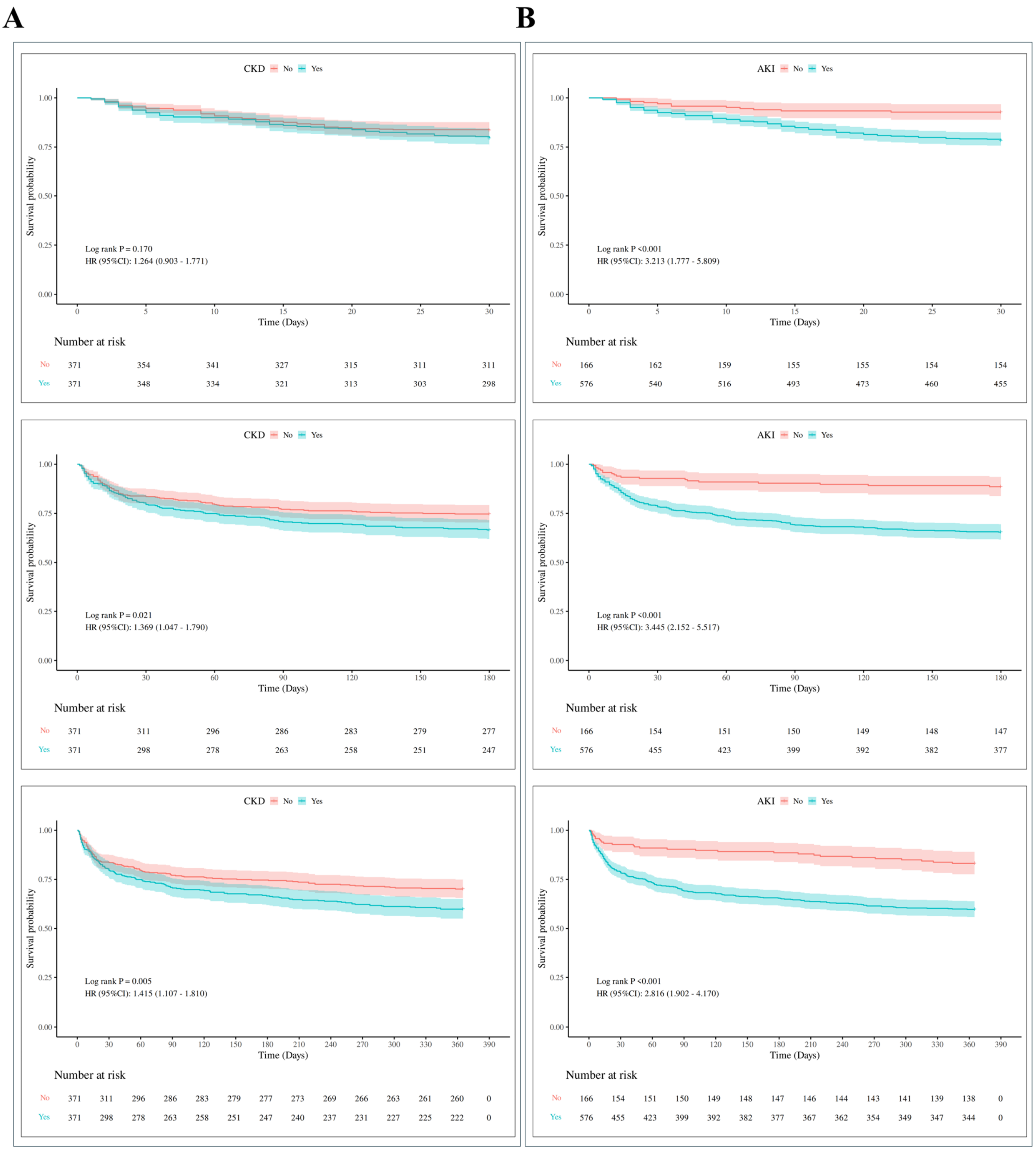

Figure S8: Survival outcomes in peripheral artery disease (PAD) patients (n=742) with or without kidney dysfunction.

Kaplan–Meier survival curves for all-cause mortality at 30, 180, and 365 days among PAD patients, stratified by the presence or absence of **(A)** chronic kidney disease (CKD) or **(B)** acute kidney injury (AKI). Statistical significance was determined using the log-rank test. The Hazard Ratios (HR) and corresponding 95% confidence intervals (CI), along with exact p values, are indicated within each panel.

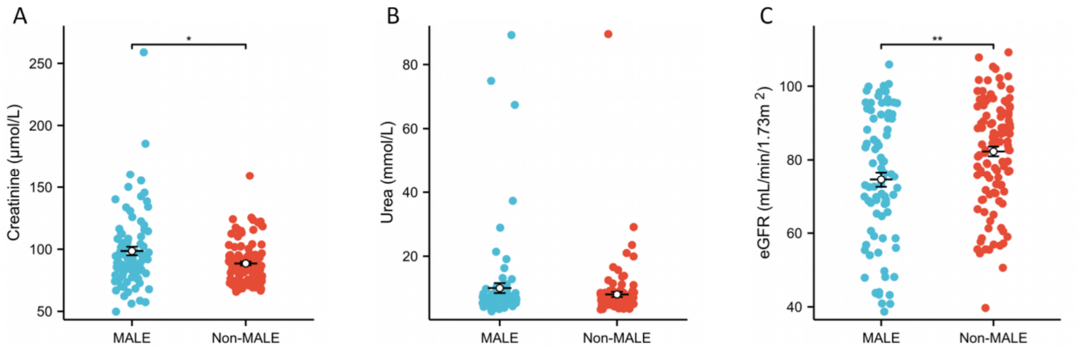

Figure S9. Comparison of renal function parameters between MALE (n=83) and Non-MALE (n=117) patients.
(A) Creatinine (µmol/L). **(B)** Urea (mmol/L). **(C)** eGFR (mL/min/1.73m²). Data are presented as median with interquartile range (IQR). Statistical significance was determined using the Mann-Whitney U test. *, $p < 0.05$ and **, $p < 0.01$.

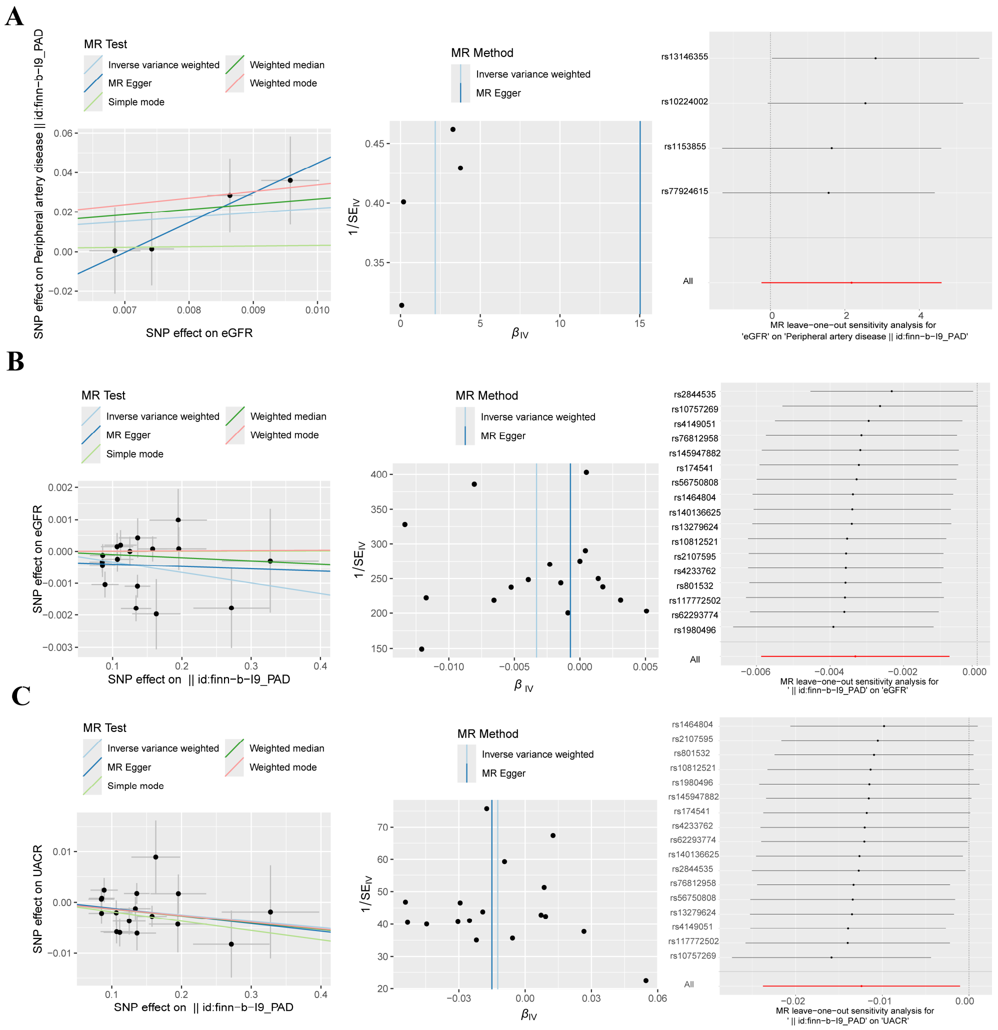

Figure S10: Sensitivity Analysis of bidirectional exposure-outcome causation.

Scatter plot for pleiotropy test, funnel plot for heterogeneity assessment, and Leave-one-out analysis of causal effect of **(A)** eGFR on PAD; **(B)** PAD on eGFR; **(C)** UACR on eGFR. PAD (Peripheral artery disease), eGFR (estimated glomerular filtration rate), UACR (urinary albumin-to-creatinine ratio), SNP (single nucleotide polymorphism).

Supplementary Tables

Table S1: Summary of genome-wide association study (GWAS) datasets utilized for Mendelian randomization analyses.

Traits	Resources	Participants	Number of SNPs	Ancestry	PMID	Author
PAD	FinnGen	7,098 cases and 206,541 controls (finn-b-I9_PAD)	9,637,467	European	NA	NA
eGFR	CKDGen	567,460	NA	European	31980069	Wuttke et al.
UACR	CKDGen	348,954 (51,861 cases and 297,093 controls)	NA	European	31980069	Teumer et al.
cfPWV	LIFE-Adult	7,669	532,676	European	32790701	Michael Rode et al.
bfPWV	LIFE-Adult	7,669	532,676	European	32790701	Michael Rode et al.
baPWV	LIFE-Adult	7,669	532,676	European	32790701	Michael Rode et al.

Abbreviations: PAD (Peripheral artery disease), eGFR (estimated glomerular filtration rate), UACR (urinary albumin-to-creatinine ratio), SNP (single nucleotide polymorphism), PWV (pulse wave velocity); cfPWV (carotid–femoral pulse wave velocity), baPWV (brachial–ankle pulse wave velocity), bfPWV (brachial–femoral pulse wave velocity).

Table S2: Trends in crude and age-adjusted mortality rates among PAD patients with CKD as a contributing cause in the United States (1999–2023)

Year	Deaths	Population	Crude Rate (95CI)	Crude Rate SE	Age Adjusted Rate (95CI)	Age Adjusted Rate SE
1999	157	180408769	0.087 (0.073-0.101)	0.007	0.093 (0.078-0.107)	0.008
2000	164	181984640	0.09 (0.076-0.104)	0.007	0.084 (0.071-0.097)	0.007
2001	188	184305128	0.102 (0.087-0.117)	0.007	0.109 (0.093-0.125)	0.008
2002	205	186208028	0.11 (0.095-0.125)	0.008	0.1 (0.086-0.115)	0.007
2003	191	188090429	0.102 (0.087-0.116)	0.007	0.107 (0.091-0.122)	0.008
2004	234	190205384	0.123 (0.107-0.139)	0.008	0.123 (0.107-0.139)	0.008
2005	207	192551384	0.108 (0.093-0.122)	0.007	0.096 (0.082-0.109)	0.007
2006	235	195019359	0.121 (0.105-0.136)	0.008	0.119 (0.103-0.134)	0.008
2007	220	197403777	0.111 (0.097-0.126)	0.008	0.098 (0.085-0.112)	0.007
2008	224	199795090	0.112 (0.097-0.127)	0.007	0.106 (0.091-0.121)	0.007
2009	235	202107016	0.116 (0.101-0.131)	0.008	0.119 (0.104-0.135)	0.008
2010	240	203891983	0.118 (0.103-0.133)	0.008	0.111 (0.097-0.126)	0.007
2011	269	206592936	0.13 (0.115-0.146)	0.008	0.126 (0.11-0.141)	0.008
2012	251	208826037	0.12 (0.105-0.135)	0.008	0.109 (0.095-0.123)	0.007
2013	238	211085314	0.113 (0.098-0.127)	0.007	0.109 (0.095-0.123)	0.007
2014	247	213809280	0.116 (0.101-0.13)	0.007	0.102 (0.089-0.115)	0.007
2015	241	216553817	0.111 (0.097-0.125)	0.007	0.097 (0.085-0.11)	0.006
2016	266	218641417	0.122 (0.107-0.136)	0.007	0.107 (0.093-0.12)	0.007
2017	237	221447331	0.107 (0.093-0.121)	0.007	0.093 (0.081-0.105)	0.006
2018	248	223311190	0.111 (0.097-0.125)	0.007	0.093 (0.081-0.105)	0.006
2019	259	224981167	0.115 (0.101-0.129)	0.007	0.093 (0.081-0.104)	0.006
2020	268	226635013	0.118 (0.104-0.132)	0.007	0.09 (0.079-0.101)	0.006
2021	332	228238412	0.145 (0.13-0.161)	0.008	0.119 (0.106-0.133)	0.007
2022	468	229508599	0.204 (0.185-0.222)	0.009	0.154 (0.14-0.169)	0.007
2023	532	231529762	0.23 (0.21-0.249)	0.010	0.179 (0.163-0.195)	0.008

Rates are expressed per 100,000 population. The 95% confidence intervals (CI) and standard errors (SE) are provided for each rate estimate. Abbreviations: PAD (Peripheral artery disease), CKD (chronic kidney disease).

Table S3: Trends in crude and age-adjusted mortality rates among PAD patients with AKI as a contributing cause in the United States (1999–2023).

Year	Deaths	Population	Crude Rate (95CI)	Crude Rate SE	Age Adjusted Rate (95CI)	Age Adjusted Rate SE
1999	157	180408769	0.087 (0.073-0.101)	0.007	0.093 (0.078-0.107)	0.008
2000	164	181984640	0.09 (0.076-0.104)	0.007	0.084 (0.071-0.097)	0.007
2001	188	184305128	0.102 (0.087-0.117)	0.007	0.109 (0.093-0.125)	0.008
2002	205	186208028	0.11 (0.095-0.125)	0.008	0.1 (0.086-0.115)	0.007
2003	191	188090429	0.102 (0.087-0.116)	0.007	0.107 (0.091-0.122)	0.008
2004	234	190205384	0.123 (0.107-0.139)	0.008	0.123 (0.107-0.139)	0.008
2005	207	192551384	0.108 (0.093-0.122)	0.007	0.096 (0.082-0.109)	0.007
2006	235	195019359	0.121 (0.105-0.136)	0.008	0.119 (0.103-0.134)	0.008
2007	220	197403777	0.111 (0.097-0.126)	0.008	0.098 (0.085-0.112)	0.007
2008	224	199795090	0.112 (0.097-0.127)	0.007	0.106 (0.091-0.121)	0.007
2009	235	202107016	0.116 (0.101-0.131)	0.008	0.119 (0.104-0.135)	0.008
2010	240	203891983	0.118 (0.103-0.133)	0.008	0.111 (0.097-0.126)	0.007
2011	269	206592936	0.13 (0.115-0.146)	0.008	0.126 (0.11-0.141)	0.008
2012	251	208826037	0.12 (0.105-0.135)	0.008	0.109 (0.095-0.123)	0.007
2013	238	211085314	0.113 (0.098-0.127)	0.007	0.109 (0.095-0.123)	0.007
2014	247	213809280	0.116 (0.101-0.13)	0.007	0.102 (0.089-0.115)	0.007
2015	241	216553817	0.111 (0.097-0.125)	0.007	0.097 (0.085-0.11)	0.006
2016	266	218641417	0.122 (0.107-0.136)	0.007	0.107 (0.093-0.12)	0.007
2017	237	221447331	0.107 (0.093-0.121)	0.007	0.093 (0.081-0.105)	0.006
2018	248	223311190	0.111 (0.097-0.125)	0.007	0.093 (0.081-0.105)	0.006
2019	259	224981167	0.115 (0.101-0.129)	0.007	0.093 (0.081-0.104)	0.006
2020	268	226635013	0.118 (0.104-0.132)	0.007	0.09 (0.079-0.101)	0.006
2021	332	228238412	0.145 (0.13-0.161)	0.008	0.119 (0.106-0.133)	0.007
2022	468	229508599	0.204 (0.185-0.222)	0.009	0.154 (0.14-0.169)	0.007
2023	532	231529762	0.23 (0.21-0.249)	0.010	0.179 (0.163-0.195)	0.008

Rates are expressed per 100,000 population. The 95% confidence intervals (CI) and standard errors (SE) are provided for each rate estimate. Abbreviations: PAD (Peripheral artery disease), AKI (acute kidney injury).

Table S4: Baseline clinical and demographic characteristics of PAD patients stratified by 180-day all-cause mortality status.

Characteristic	Overall<br>N = 742	180d survivor<br>N = 524	180d mortality<br>N = 218	*P*-value
Age, Median (Q1, Q3)	73.00 (66.00, 81.00)	71.00 (64.00, 78.00)	78.00 (70.00, 84.00)	<0.001
Gender, n (%)				0.141
Female	303 (41%)	205 (39%)	98 (45%)	
Male	439 (59%)	319 (61%)	120 (55%)	
Race, n (%)				0.081
African	42 (6%)	32 (6%)	10 (5%)	
Other	207 (28%)	134 (26%)	73 (33%)	
Caucasion	493 (66%)	358 (68%)	135 (62%)	
SOFA, Median (Q1, Q3)	5.00 (3.00, 7.00)	4.00 (2.00, 6.00)	6.00 (4.00, 9.00)	<0.001
Heart rate, Median (Q1, Q3)	83.00 (73.00, 96.00)	81.00 (71.00, 93.00)	89.00 (75.00, 103.00)	<0.001
SpO_2, Median (Q1, Q3)	98.00 (95.00, 100.00)	98.00 (96.00, 100.00)	97.00 (94.00, 100.00)	<0.001
SBP, Median (Q1, Q3)	121.00 (105.00, 138.00)	123.00 (106.50, 140.00)	117.00 (101.00, 136.00)	0.008
DBP, Median (Q1, Q3)	61.00 (52.00, 74.00)	61.00 (52.00, 74.00)	62.00 (52.00, 75.00)	0.756
MBP, Median (Q1, Q3)	79.00 (69.00, 91.00)	80.00 (70.00, 91.00)	77.00 (68.00, 92.00)	0.235
RR, Median (Q1, Q3)	18.00 (15.00, 22.00)	17.00 (14.00, 21.00)	20.00 (16.00, 24.00)	<0.001
Heart failure, n (%)	408 (55%)	261 (50%)	147 (67%)	<0.001
AS, n (%)	353 (48%)	248 (47%)	105 (48%)	0.835
Diabetes, n (%)	288 (39%)	192 (37%)	96 (44%)	0.060
Hypertension, n (%)	162 (22%)	107 (20%)	55 (25%)	0.149
AKI, n (%)	576 (78%)	377 (72%)	199 (91%)	<0.001
AKI stage, n (%)				<0.001
No	166 (22%)	147 (28%)	19 (9%)	
1	145 (20%)	111 (21%)	34 (16%)	
2	262 (35%)	198 (38%)	64 (29%)	
3	169 (23%)	68 (13%)	101 (46%)	
RBC, Median (Q1, Q3)	3.57 (3.13, 4.06)	3.56 (3.15, 4.06)	3.60 (3.04, 4.02)	0.342
WBC, Median (Q1, Q3)	9.70 (7.20, 13.70)	9.40 (7.20, 13.10)	10.65 (7.80, 14.30)	0.008
Platelet, Median (Q1, Q3)	198.00 (154.00, 260.00)	198.00 (157.00, 256.00)	196.50 (139.00, 264.00)	0.498
Glucose, Median (Q1, Q3)	126.00 (101.00, 164.00)	123.00 (100.00, 159.00)	131.00 (101.00, 182.00)	0.144
BUN, Median (Q1, Q3)	25.00 (17.00, 41.00)	23.00 (17.00, 35.50)	34.00 (22.00, 54.00)	<0.001
SCr, Median (Q1, Q3)	1.20 (0.90, 1.80)	1.10 (0.85, 1.60)	1.40 (1.00, 2.20)	<0.001
Urineoutput, Median (Q1, Q3)	145.00 (55.00, 300.00)	150.00 (60.00, 300.00)	125.00 (50.00, 250.00)	0.071
MV, n (%)	654 (88%)	448 (85%)	206 (94%)	<0.001
CRRT, n (%)	57 (8%)	19 (4%)	38 (17%)	<0.001
CKD, n (%)	371 (50%)	247 (47%)	124 (57%)	0.016
CKD stage, n (%)				<0.001
No	371 (50%)	277 (53%)	94 (43%)	
1-3	252 (34%)	180 (34%)	72 (33%)	
4-5	119 (16%)	67 (13%)	52 (24%)	
eGFR MDRD, Median (Q1, Q3)	55.31 (34.71, 78.52)	58.05 (39.45, 84.43)	44.49 (28.04, 66.57)	<0.001
Time 30d, Median (Q1, Q3)	30.00 (30.00, 30.00)	30.00 (30.00, 30.00)	18.00 (7.00, 30.00)	<0.001
Death 30d, n (%)	137 (18%)	0 (0%)	137 (63%)	<0.001
Time 180d, Median (Q1, Q3)	180.00 (84.00, 180.00)	180.00 (180.00, 180.00)	18.00 (7.00, 55.00)	<0.001
Time 365d, Median (Q1, Q3)	365.00 (84.00, 365.00)	365.00 (365.00, 365.00)	18.00 (7.00, 55.00)	<0.001
Death 365d, n (%)	260 (35%)	42 (8%)	218 (100%)	<0.001

Data are presented as median (Q1, Q3) for continuous variables and as n (%) for categorical variables. Statistical comparisons between the 180-day survivor (n = 524) and 180-day mortality (n = 218) groups were performed using the Mann-Whitney U test for continuous variables and Pearson’s χ^2 test (or Fisher's exact test, as appropriate) for categorical variables. The biological units represent individual human patients (total n = 742). Exact p values are provided within the table; $p < 0.001$ indicates values below the limit of precision.

Abbreviations: PAD, Peripheral Artery Disease; CKD, Chronic Kidney Disease; AKI, Acute Kidney Injury; SOFA, Sequential Organ Failure Assessment; SBP, Systolic Blood Pressure; DBP, Diastolic Blood Pressure; MBP, Mean Blood Pressure; RR, Respiratory Rate; SpO_2, Peripheral Capillary Oxygen Saturation; RBC, Red Blood Cell count; WBC, White Blood Cell count; BUN, Blood Urea Nitrogen; SCr, Serum Creatinine; MV, Mechanical Ventilation; CRRT, Continuous Renal Replacement Therapy; eGFR MDRD, Estimated Glomerular Filtration Rate calculated by the Modification of Diet in Renal Disease equation; Q1, First Quartile; Q3, Third Quartile.

Table S5: Univariate Cox proportional hazards regression analysis of potential baseline risk factors for 180-day all-cause mortality in PAD patients.

**Variables**	***P***	**HR (95%CI)**
Gender		
Female		1.00 (Reference)
Male	0.164	0.83 (0.63 ~ 1.08)
Race		
African		1.00 (Reference)
Other	0.130	1.67 (0.86 ~ 3.23)
Caucasian	0.637	1.17 (0.61 ~ 2.22)
Age	<.001	1.05 (1.03 ~ 1.06)
SOFA	<.001	1.16 (1.13 ~ 1.20)
Heart rate	<.001	1.02 (1.01 ~ 1.02)
SpO_2	<.001	0.96 (0.94 ~ 0.98)
SBP	0.022	0.99 (0.99 ~ 0.99)
RR	<.001	1.05 (1.03 ~ 1.07)
Heart failure		
No		1.00 (Reference)
Yes	<.001	1.81 (1.36 ~ 2.40)
AKI		
No		1.00 (Reference)
Yes	<.001	3.45 (2.15 ~ 5.52)
AKI stage		
No		1.00 (Reference)
1	0.007	2.18 (1.24 ~ 3.82)
2	0.002	2.28 (1.37 ~ 3.81)
3	<.001	7.31 (4.47 ~ 11.94)
WBC	<.001	1.03 (1.01 ~ 1.05)
BUN	<.001	1.01 (1.01 ~ 1.02)
SCr	0.036	1.08 (1.01 ~ 1.15)
MV		
No		1.00 (Reference)
Yes	0.001	2.64 (1.47 ~ 4.72)
CRRT		
No		1.00 (Reference)
Yes	<.001	3.49 (2.46 ~ 4.96)
eGFR MDRD	<.001	0.99 (0.99 ~ 0.99)

Statistical significance and effect sizes were determined using univariate Cox proportional hazards regression models. The biological units represent individual human patients (total n = 742). Hazard ratios (HR) and corresponding 95% confidence intervals (CI) are presented for each baseline variable. Exact p values are provided within the table; $p < 0.001$ indicates

values below the limit of precision.

Abbreviations: PAD, Peripheral Artery Disease; HR, Hazard Ratio; CI, Confidence Interval; SOFA, Sequential Organ Failure Assessment; SpO_2, Peripheral Capillary Oxygen Saturation; SBP, Systolic Blood Pressure; RR, Respiratory Rate; AKI, Acute Kidney Injury; WBC, White Blood Cell count; BUN, Blood Urea Nitrogen; SCr, Serum Creatinine; MV, Mechanical Ventilation; CRRT, Continuous Renal Replacement Therapy; eGFR MDRD, Estimated Glomerular Filtration Rate calculated using the Modification of Diet in Renal Disease equation.

Table S6: Univariate Cox proportional hazards regression analysis of potential baseline risk factors for 365-day all-cause mortality in patients with PAD.

Variables	β	S.E	Z	*P*	HR (95%CI)
Gender					
Female					1.00 (Reference)
Male	-0.20	0.12	-1.62	0.105	0.82 (0.64 ~ 1.04)
Race					
African					1.00 (Reference)
Other	0.35	0.29	1.20	0.229	1.41 (0.80 ~ 2.49)
Caucasian	-0.01	0.28	-0.03	0.978	0.99 (0.57 ~ 1.71)
AKI					
No					1.00 (Reference)
Yes	1.04	0.20	5.17	<.001	2.82 (1.90 ~ 4.17)
CKD					
No					1.00 (Reference)
Yes	0.35	0.13	2.77	0.006	1.42 (1.11 ~ 1.81)
CKD stage					
0					1.00 (Reference)
1-3	0.16	0.14	1.14	0.255	1.18 (0.89 ~ 1.56)
4-5	0.67	0.16	4.25	<.001	1.96 (1.43 ~ 2.66)
Heart failure					
No					1.00 (Reference)
Yes	0.63	0.13	4.77	<.001	1.88 (1.45 ~ 2.44)
Diabetes					
No					1.00 (Reference)
Yes	0.21	0.13	1.65	0.098	1.23 (0.96 ~ 1.57)
AS					
No					1.00 (Reference)
Yes	0.09	0.12	0.76	0.447	1.10 (0.86 ~ 1.40)
Hypertension					
No					1.00 (Reference)
Yes	0.15	0.14	1.04	0.299	1.16 (0.88 ~ 1.54)
AKI stage					
No					1.00 (Reference)
1	0.57	0.25	2.31	0.021	1.77 (1.09 ~ 2.86)
2	0.71	0.22	3.23	0.001	2.03 (1.32 ~ 3.11)
3	1.77	0.21	8.31	<.001	5.85 (3.86 ~ 8.87)
MV					

No					1.00 (Reference)
Yes	0.81	0.25	3.21	0.001	2.24 (1.37 ~ 3.66)
CRRT					
No					1.00 (Reference)
Yes	1.20	0.17	7.03	<.001	3.33 (2.38 ~ 4.65)
SOFA	0.14	0.02	9.05	<.001	1.16 (1.12 ~ 1.19)
Age	0.04	0.01	6.50	<.001	1.04 (1.03 ~ 1.05)
Heart rate	0.01	0.00	4.62	<.001	1.01 (1.01 ~ 1.02)
SpO_2	-0.04	0.01	-4.50	<.001	0.96 (0.94 ~ 0.98)
SBP	-0.01	0.00	-2.64	0.008	0.99 (0.99 ~ 0.99)
DBP	0.00	0.00	0.51	0.607	1.00 (0.99 ~ 1.01)
MBP	-0.01	0.00	-1.41	0.158	0.99 (0.99 ~ 1.00)
RR	0.05	0.01	6.30	<.001	1.05 (1.03 ~ 1.07)
RBC	-0.11	0.09	-1.22	0.224	0.90 (0.75 ~ 1.07)
WBC	0.03	0.01	3.58	<.001	1.03 (1.01 ~ 1.05)
Platelet	-0.00	0.00	-0.34	0.733	1.00 (1.00 ~ 1.00)
Glucose	0.00	0.00	1.65	0.098	1.00 (1.00 ~ 1.00)
BUN	0.01	0.00	7.21	<.001	1.01 (1.01 ~ 1.02)
Creatinine	0.10	0.03	3.41	<.001	1.10 (1.04 ~ 1.17)
Urine output	-0.01	0.00	-2.03	0.042	0.99 (0.99 ~ 0.99)
EGFR mdrd	-0.01	0.00	-4.81	<.001	0.99 (0.99 ~ 0.99)

Statistical significance and effect sizes were determined using univariate Cox proportional hazards regression models. The biological units represent individual human patients (total n = 742). Hazard ratios (HR) and corresponding 95% confidence intervals (CI) are presented for each baseline variable. Exact p values are provided within the table; p < 0.001 indicates values below the limit of precision.

Abbreviations: PAD, Peripheral Artery Disease; HR, Hazard Ratio; CI, Confidence Interval; AKI, Acute Kidney Injury; CKD, Chronic Kidney Disease; AS, Atherosclerosis; SOFA, Sequential Organ Failure Assessment; SBP, Systolic Blood Pressure; DBP, Diastolic Blood Pressure; MBP, Mean Blood Pressure; RR, Respiratory Rate; SpO_2, Peripheral Capillary Oxygen Saturation; RBC, Red Blood Cell count; WBC, White Blood Cell count; BUN, Blood Urea Nitrogen; Creatinine, Serum Creatinine; MV, Mechanical Ventilation; CRRT, Continuous Renal Replacement Therapy; eGFR MDRD, Estimated Glomerular Filtration Rate calculated using the Modification of Diet in Renal Disease equation.

Table S7. Distribution of major adverse limb events (MALE) across estimated glomerular filtration rate (eGFR) categories in patients with PAD.

eGFR category (mL/min/1.73 m²)	MALE, n (%)	Non-MALE, n (%)
≥ 90	23 (27.7)	38 (32.5)
75 to < 90	18 (21.7)	48 (41.0)
60 to < 75	22 (26.5)	19 (16.2)
30 to < 60	20 (24.1)	12 (10.3)

Data are presented as n (%). The biological units represent individual human patients (total n = 200, comprising 83 in the MALE group and 117 in the Non-MALE group).

Abbreviations: MALE, major adverse limb events.

Table S8: Stepwise selection and filtering criteria for single nucleotide polymorphisms (SNPs) utilized as instrumental variables in bidirectional Mendelian randomization analyses.

Step	Filtering step	Criteria	eGFR → PAD	UACR → PAD	PAD → UACR	PAD → eGFR
1	Original GWAS instruments	Genome-wide significant SNPs ($p < 5 \times 10^{-6}$)	45,739	8,078	-	-
2	LD clumping	$r^2 < 0.001$, window = 10,000 kb	332	137	23	23
3	Minor allele frequency filter	EAF > 0.10	332	137	20	20
4	Instrument strength	F-statistic > 10	4	137	20	20
5	Multivariable adjustment	Adjusted for smoking, alcohol use, and BMI	4	137	20	20
6	Outcome data extraction	SNPs available in outcome GWAS	4	0	18	18
7	Harmonisation	Allele alignment between exposure and outcome	4	-	17	17
8	Palindromic SNP exclusion	Removed palindromic SNPs with intermediate allele frequencies	4	-	17	17

Abbreviations: MR, Mendelian randomization; SNP, single-nucleotide polymorphism; GWAS, genome-wide association study; eGFR, estimated glomerular filtration rate; UACR, urinary albumin-to-creatinine ratio; PAD, peripheral artery disease; EAF, effect allele frequency; LD, linkage disequilibrium; BMI, body mass index.

Table S9: Detailed characteristics and summary statistics of instrumental variables (SNPs) used in the Mendelian randomization analysis assessing the causal effect of eGFR on PAD.

	SNP	Effect allele	Other allele	Chromosome	Position	Exposure (eGFR)				Outcome (PAD)				r^2	F
						Eaf	Beta	SE	p value	Eaf	Beta	SE	p value		
1	rs10224002	A	G	7	151415041	0.7200	0.0068	0.0004	2.737e-66	0.7658	5e-04	0.0218	0.9833	1.89E-05	10.3402
2	rs1153855	C	G	15	45660758	0.6200	0.0086	0.0004	1.23e-132	0.5868	0.0284	0.0187	0.1285	3.51E-05	19.9421
3	rs13146355	A	G	4	77412140	0.4400	-0.0074	0.0003	8.31e-102	0.4558	-0.0014	0.0185	0.9392	1.00E+00	15.2397
4	rs77924615	A	G	16	20392332	0.2000	0.0096	0.0005	1.21e-99	0.2223	0.0360	0.0223	0.1066	2.00E+00	15.3825

Effect sizes (Beta) and standard errors (SE) are provided for each single nucleotide polymorphism (SNP) alongside their exact p values. The proportion of variance in the exposure explained by each instrument (r^2) and the F-statistic (indicating instrument strength) are also reported.

Abbreviations: MR: Mendelian randomization; SNP: Single nucleotide polymorphism; PAD: Peripheral artery disease; eGFR: estimated glomerular filtration rate.

Table S10: Detailed characteristics and summary statistics of instrumental variables (SNPs) used in the Mendelian randomization analysis assessing the causal effect of PAD on eGFR.

	SNP	Effect allele	Other allele	Chromosome	Position	Exposure (PAD)				Outcome (eGFR)				r^2	F
						Eaf	Beta	SE	p value	Eaf	Beta	SE	p value		
1	rs10757269	G	A	9	22072264	0.442	0.1359	0.0184	1.6761e-13	0.48	-0.001095	0.003526	0.001897	9.11E-03	1963.9743
2	rs10812521	A	G	9	27051812	0.1909	-0.125	0.0235	1.035e-07	0.17	0	0.004543	0.9998	4.83E-03	1036.0848
3	rs117772502	A	G	13	98393867	0.05798	-0.1955	0.0399	9.52796e-07	0.081	-8.20E-05	0.006735	0.9036	4.18E-03	895.6012
4	rs13279624	A	G	8	24368386	0.5296	-0.0857	0.0184	3.41901e-06	0.52	0.000125	0.003514	0.723	3.66E-03	784.5758
5	rs140136625	T	C	12	76569281	0.01858	0.3276	0.0698	2.715e-06	0.018	-0.000299	0.016356	0.8551	3.91E-03	839.3776
6	rs145947882	C	A	17	41809207	0.02994	0.2715	0.0548	7.34007e-07	0.03	-0.00178	0.00124	0.1511	4.28E-03	918.5851
7	rs1464804	A	T	7	17960827	0.1977	0.107	0.023	3.287e-06	0.26	-0.000246	0.003954	0.5343	3.63E-03	778.6764
8	rs174541	C	T	11	61565908	0.4447	-0.0854	0.0185	3.73697e-06	0.37	0.000448	0.003594	0.2126	3.60E-03	772.2244
9	rs1980496	T	C	6	32340070	0.2975	0.1578	0.0211	8.16582e-14	0.37	7.8e-05	0.003916	0.8429	1.04E-02	2246.7618
10	rs2107595	A	G	7	19049388	0.1942	0.1115	0.0232	1.46899e-06	0.16	0.000195	0.004683	0.6768	3.89E-03	834.4208
11	rs2844535	G	T	6	31350303	0.2689	0.1339	0.0218	8.52001e-10	0.26	-0.001788	0.004085	1.212e-05	7.05E-03	1516.5857
12	rs4149051	G	A	12	21331057	0.3387	-0.0891	0.0195	4.74799e-06	0.25	0.001045	0.004006	0.00911	3.56E-03	762.3987
13	rs4233762	G	A	2	19302855	0.7997	0.1065	0.0232	4.26197e-06	0.79	0.000149	0.004261	0.7258	3.63E-03	779.0297
14	rs56750808	C	T	18	74676704	0.4557	0.0851	0.0185	4.14601e-06	0.49	-0.000335	0.003425	0.3277	3.59E-03	770.2032

15	rs62293774	A	C	4	21016091	0.05234	-0.1947	0.0414	2.56201e-06	0.042	-0.000989	0.000958 6	0.3024	3.76E-03	806.3438
16	rs76812958	G	T	9	10579595	0.07507	0.1631	0.0349	3.07001e-06	0.03	-0.001969	0.001094	0.07185	3.69E-03	792.0568
17	rs801532	G	A	7	18661156	0.8695	0.1362	0.0273	6.29695e-07	0.9	0.000423	0.000621 6	0.4957	4.21E-03	903.0926

Effect sizes (Beta) and standard errors (SE) are provided for each single nucleotide polymorphism (SNP) alongside their exact p values. The proportion of variance in the exposure explained by each instrument (r^2) and the F-statistic (indicating instrument strength) are also reported.

Abbreviations: MR: Mendelian randomization; SNP: Single nucleotide polymorphism; PAD: Peripheral artery disease; eGFR: estimated glomerular filtration rate.

Table S11: Detailed characteristics and summary statistics of instrumental variables (SNPs) used in the Mendelian randomization analysis assessing the causal effect of PAD on UACR.

	SNP	Effect allele	Other allele	Chromosome	Position	Exposure (PAD) Eaf	Exposure (PAD) Beta	Exposure (PAD) SE	Exposure (PAD) p value	Outcome (UACR) Eaf	Outcome (UACR) Beta	Outcome (UACR) SE	Outcome (UACR) p value	r^2	F
1	rs10757269	G	A	9	22072264	0.442	0.1359	0.0184	1.68E-13	0.484	0.0017166	0.00201888	0.3952	9.11E-03	1963.9743
2	rs10812521	A	G	9	27051812	0.1909	-0.125	0.0235	1.04E-07	0.1641	0.0036763	0.00268862	0.1711	4.83E-03	1036.0848
3	rs117772502	A	G	13	98393867	0.05798	-0.1955	0.0399	9.53E-07	0.0787	-0.0016828	0.00381411	0.6591	4.18E-03	895.6012
4	rs13279624	A	G	8	24368386	0.5296	-0.0857	0.0184	3.42E-06	0.5144	-0.0007929	0.002022	0.6949	3.66E-03	784.5758
5	rs140136625	T	C	12	76569281	0.01858	0.3276	0.0698	2.72E-06	0.016	-0.0018719	0.00917951	0.8384	3.91E-03	839.3776
6	rs145947882	C	A	17	41809207	0.02994	0.2715	0.0548	7.34E-07	0.0262	-0.0082562	0.006661	0.2152	4.28E-03	918.5851
7	rs1464804	A	T	7	17960827	0.1977	0.107	0.023	3.29E-06	0.2531	-0.0057958	0.00228873	0.01128	3.63E-03	778.6764
8	rs174541	C	T	11	61565908	0.4447	-0.0854	0.0185	3.74E-06	0.3573	0.0021499	0.00207911	0.3011	3.60E-03	772.2244
9	rs1980496	T	C	6	32340070	0.2975	0.1578	0.0211	8.17E-14	0.4183	-0.0027455	0.002084	0.1877	1.04E-02	2246.7618
10	rs2107595	A	G	7	19049388	0.1942	0.1115	0.0232	1.47E-06	0.1555	-0.0059333	0.00274999	0.03096	3.89E-03	834.4208
11	rs2844535	G	T	6	31350303	0.2689	0.1339	0.0218	8.52E-10	0.2703	-0.0012494	0.00225779	0.58	7.05E-03	1516.5857
12	rs4149051	G	A	12	21331057	0.3387	-0.0891	0.0195	4.75E-06	0.2289	-0.0023718	0.00236166	0.3152	3.56E-03	762.3987
13	rs4233762	G	A	2	19302855	0.7997	0.1065	0.0232	4.26E-06	0.7856	-0.002044	0.00243177	0.4006	3.63E-03	779.0297
14	rs56750808	C	T	18	74676704	0.4557	0.0851	0.0185	4.15E-06	0.489	0.0006137	0.00198711	0.7574	3.59E-03	770.2032

15	rs62293774	A	C	4	21016091	0.05234	-0.1947	0.0414	2.56E-06	0.0365	0.0042955	0.0055493	0.4389	3.76E-03	806.3438
16	rs76812958	G	T	9	10579595	0.07507	0.1631	0.0349	3.07E-06	0.0199	0.0089201	0.0072654	0.2195	3.69E-03	792.0568
17	rs801532	G	A	7	18661156	0.8695	0.1362	0.0273	6.30E-07	0.9009	-0.0060729	0.0034027	0.07431	4.21E-03	903.0926

Effect sizes (Beta) and standard errors (SE) are provided for each single nucleotide polymorphism (SNP) alongside their exact p values. The proportion of variance in the exposure explained by each instrument (r^2) and the F-statistic (indicating instrument strength) are also reported.

Abbreviations: MR: Mendelian randomization; SNP: Single nucleotide polymorphism; PAD: Peripheral artery disease; UACR: urinary albumin-to-creatinine ratio.

Table S12: Mendelian randomization estimates and corresponding sensitivity analyses for the causal effects of kidney function indicators (eGFR and UACR) on pulse wave velocity (PWV) traits.

	Exposure	Outcome	nSNP	β	SE	pval	Egger_intercept	SE_intercept	pval_intercept	Q	Q_df	Q_pval
1	eGFR	cfPWV	4	0.2146	0.3003	0.4750	-0.0212	0.0191	0.3825	3.2556	3	0.353871
2	eGFR	bfPWV	4	-0.0693	0.4870	0.8869	-0.0060	0.0322	0.8701	0.9038	3	0.824521
3	eGFR	baPWV	4	0.1182	0.1354	0.3826	0.0015	0.0089	0.8827	0.2308	3	0.972475
4	UACR	cfPWV	105	-0.0268	0.0337	0.4263	-0.0005	0.0014	0.6975	79.8689	104	0.962162
5	UACR	bfPWV	105	0.0664	0.0617	0.2823	-0.0029	0.0025	0.2450	122.6870	104	0.101876
6	UACR	baPWV	105	-0.0154	0.0177	0.3822	-0.0008	0.0007	0.2622	130.0132	104	0.042954

Effect sizes (Beta) and standard errors (SE) are provided for each single nucleotide polymorphism (SNP) alongside their exact p values. The proportion of variance in the exposure explained by each instrument (r^2) and the F-statistic (indicating instrument strength) are also reported.

Abbreviations: eGFR (estimated glomerular filtration rate), UACR (urinary albumin-to-creatinine ratio), SNP (single nucleotide polymorphism), PWV (pulse wave velocity); cfPWV (carotid–femoral pulse wave velocity), baPWV (brachial–ankle pulse wave velocity), bfPWV (brachial–femoral pulse wave velocity).

Table S13: List of abbreviations used in the study.

Abbreviation	Full Name
PAD	Peripheral artery disease
CKD	Chronic kidney disease
AKI	Acute kidney injury
GBD	Global Burden of Disease
CDC WONDER	Centers for Disease Control and Prevention Wide-ranging Online Data for Epidemiologic Research
MIMIC-IV	Medical Information Mart for Intensive Care IV
2H-SXMU	Second Hospital of Shanxi Medical University
MR	Mendelian randomization
SDI	Socio-demographic index
ASPR	Age-standardized prevalence rate
ASDR	Age-standardized death rate
ASDALYR	Age-standardized disability-adjusted life year rate
PAF	Population-attributable fraction
NCHS	National Center for Health Statistics
ICD-10	International Classification of Diseases, Tenth Revision
AAMR	Age-adjusted mortality rate
SpO_2	Oxygen saturation
RR	Respiratory rate
SBP	Systolic blood pressure
DBP	Diastolic blood pressure
MAP	Mean arterial pressure
MV	Mechanical ventilation
CRRT	Continuous renal replacement therapy
PSM	Propensity score matching
HR	Hazard ratio
CI	Confidence interval
UI	Uncertainty interval
MALE	Major adverse limb events
SNP	Single nucleotide polymorphism
IV	Instrumental variable
eGFR	Estimated glomerular filtration rate
GWAS	Genome-wide association study
PWV	Pulse wave velocity
cfPWV	Carotid–femoral pulse wave velocity
baPWV	Brachial–ankle pulse wave velocity
bfPWV	Brachial–femoral pulse wave velocity
LD	Linkage disequilibrium
IVW	Inverse-variance weighted
WME	Weighted median estimator
MR-ER	MR-Egger regression
ABI	Ankle-brachial index
